# CircZNF215 promotes tumor growth and metastasis through inactivation of the PTEN/AKT pathway in intrahepatic cholangiocarcinoma

**DOI:** 10.1186/s13046-023-02699-w

**Published:** 2023-05-18

**Authors:** Wenwei Liao, Jinpeng Du, Lian Li, Xianquan Wu, Xing Chen, Qingbo Feng, Lin Xu, Xiangzheng Chen, Mingheng Liao, Jiwei Huang, Kefei Yuan, Yong Zeng

**Affiliations:** 1 Division of Liver Surgery, Department of General Surgery, West China Hospital, Sichuan University and Collaborative Innovation Center of Biotherapy, Chengdu, 610041 China; 2 Department of Hepatobiliary and Pancreatic Surgery, The First Affiliated Hospital of Zhengzhou University, Zhengzhou, 450052 Henan China; 3 Department of General Surgery, The Fifth Affiliated Hospital of Sun Yat-sen University, Zhuhai, 519000 Guangdong China

**Keywords:** Intrahepatic cholangiocarcinoma, Metastasis, Circular RNAs, PTEN

## Abstract

**Background:**

Increasing evidence shows that circular RNAs (circRNAs), a novel class of noncoding RNAs, play a crucial role in the development of cancers, including intrahepatic cholangiocarcinoma (iCCA). Nevertheless, their functions and exact mechanisms in iCCA progression and metastasis are still unclear. Ipatasertib is a highly selective inhibitor of AKT that inhibits tumor growth by blocking the PI3K/AKT pathway. In addition, phosphatase and tensin homolog (PTEN) can also inhibit the activation of the PI3K/AKT pathway, but it is not clear whether the cZNF215-PRDX-PTEN axis plays a role in the antitumor activity of ipatasertib.

**Methods:**

We identified a new circRNA (circZNF215, termed cZNF215) through high-throughput circRNA sequencing (circRNA-seq). In addition, RT‒qPCR, immunoblot assay, RNA pull-down assay, RNA immunoprecipitation (RIP) assay, and fluorescence in situ hybridization assay (FISH) were used to investigate the interaction of cZNF215 with peroxiredoxin 1 (PRDX1). Coimmunoprecipitation (Co-IP) assays and duolink in situ proximity ligation assays (PLAs) were conducted to analyze the effects of cZNF215 on the interaction between PRDX1 and PTEN. Finally, we tested the potential effects of cZNF215 on the antitumor activity of ipatasertib with *in vivo* experiments.

**Results:**

We found that cZNF215 expression was obviously upregulated in iCCA tissues with postoperative metastases and was correlated with iCCA metastasis and poor outcome in patients with iCCA. We further revealed that overexpression of cZNF215 promoted iCCA cell growth and metastasis in vitro and in vivo, while cZNF215 knockdown had the opposite effect. Mechanistic studies suggested that cZNF215 competitively interacted with PRDX1, which blocked the association between PRDX1 and PTEN, subsequently leading to oxidation-induced inactivation of the PTEN/AKT pathway and finally contributing to iCCA progression and metastasis. Additionally, we also revealed that silencing cZNF215 in iCCA cells had the potential to enhance the antitumor effect of ipatasertib.

**Conclusions:**

Our study demonstrates that cZNF215 facilitates iCCA progression and metastasis by regulating the PTEN/AKT pathway and may serve as a novel prognostic predictor in patients with iCCA.

**Supplementary Information:**

The online version contains supplementary material available at 10.1186/s13046-023-02699-w.

## Introduction

ICCA is the second most common primary liver malignancy with an adverse prognosis [1, 2]. The incidence of iCCA has been steadily increasing globally over the past 30 years [3–5]. Although its incidence is relatively low, it is often difficult to diagnose early due to the lack of specific symptoms. Currently, surgical approaches remain the most potentially effective treatment for iCCA, but the 5-year overall survival rate is less than 40% [6–8], and postoperative recurrence rates may be as high as 65% [9, 10]. Moreover, in patients with unresectable iCCA, chemotherapy/radiotherapy is less effective [11]. In resectable iCCA, chemotherapy/radiotherapy is effective and provides a clear benefit for the majority of patients (especially in neoadjuvancy); however, its efficacy is short-lived, and patients eventually develop resistance to therapy and relapse [12]. Thus, there is an urgent need to improve our understanding of the mechanisms of iCCA progression and metastasis so that more advanced diagnostic techniques and novel therapeutic targets can be developed to improve the survival rate of iCCA patients.

CircRNAs are generated by back-splicing and form a covalently closed continuous loop without 5’ capping and 3’ polyadenylation [13, 14]. This special structure renders circRNAs particularly resistant to RNase degradation and more stable [15]. Hence, circRNAs have the potential to serve as biomarkers based on their stability [16, 17]. Currently, accumulating evidence has demonstrated that numerous dysregulated circRNAs are vital players in tumor development. These circRNAs exert their functions through various mechanisms, including by acting as microRNA (miRNA) sponges, binding to RNA-associated proteins, activating or inactivating cancer-related signaling pathways, and regulating proteins and peptides [18–20]. The underlying mechanisms of circRNAs in iCCA metastasis, however, remain to be further elucidated.

*PTEN* is the first identified tumor suppressor gene with phosphatase function and is often dysregulated in cancers. Studies have found that loss of PTEN function leads to excessive activation of the PI3K/AKT pathway, which promotes cancer progression [21–23]. Reactive oxygen species (ROS), including superoxide (O_2_^-^) and hydrogen peroxide (H_2_O_2_), are inevitably generated during aerobic and anaerobic metabolism [24], and they can activate downstream signaling via the oxidation-induced inactivation of PTEN [25, 26]. Currently, it is widely recognized that PTEN oxidation leads to loss of its phosphatase activity, sequentially activating the PI3K/AKT pathway and promoting tumorigenesis [25–27]. PRDX1, a member of the peroxiredoxin (PRDX) family, has been demonstrated to preserve and boost the tumor inhibitory function of PTEN by binding PTEN and preventing PTEN oxidation under benign oxidative stress [27, 28]. Nevertheless, it is not clear whether circRNAs regulate the PTEN/AKT pathway via PRDX1.

In this study, through circRNA sequencing profiling, we identified cZNF215 as a critical circRNA that is highly upregulated in iCCA tissues with postoperative metastases. Abnormal expression of cZNF215 promotes iCCA progression and metastasis both in vitro and in vivo by inactivation of the PTEN/AKT pathway. Specifically, cZNF215 competitively binds to PRDX1. As a result, PTEN is no longer protected by PRDX1 and is oxidatively inactivated, leading to AKT phosphorylation. More importantly, we demonstrate the possibility of cZNF215 as a therapeutic target for iCCA progression and metastasis.

## Materials and methods

### Patient samples

Patient samples, including 120 primary iCCA tissues, were obtained between 2009 and 2018 from West China Hospital. Of these, 15 iCCA tissues from patients with extrahepatic metastases after surgery and 15 iCCA tissues from patients without postoperative metastases were selected for circRNA-seq. The Institutional Ethics Committee of West China Hospital granted approval for the use of human subjects. Signed informed consent was obtained from patients in this study.

### Cell lines and reagents

All cell lines (HuCCT1, RBE and HCCC9810) were grown in RPMI 1640 medium (Gibco) with 10% fetal bovine serum (Gibco) under appropriate conditions (37°C, 5% CO_2_).

### Statistical analysis

All data are presented as the mean ± standard deviation (SD). Comparisons were performed using Student’s t test, one-way ANOVA, or Mann‒Whitney U test, as appropriate. Pearson’s correlation coefficient analysis was used to analyze the correlations between cZNF215 and p-AKT expression. Univariate and multivariate analyses were performed to analyze the relationship between iCCA patient characteristics and cZNF215 expression. The Kaplan‒Meier method and the log-rank test were applied to estimate the survival rate and the differences, respectively. GraphPad Prism 9.0 (GraphPad, USA) and SPSS 26.0 (SPSS, USA) were used for data analyses. *P*<0.05 indicated statistical significance. *P values less than 0.05, **P values less than 0.01, ***P values less than 0.001.

For more detailed methodology, please refer to the supporting materials and methods.

## Results

### CircZNF215 is considerably increased in iCCA tissues, and high expression of cZNF215 is correlated with metastasis and poor prognosis in iCCA patients

**Fig. 1:**
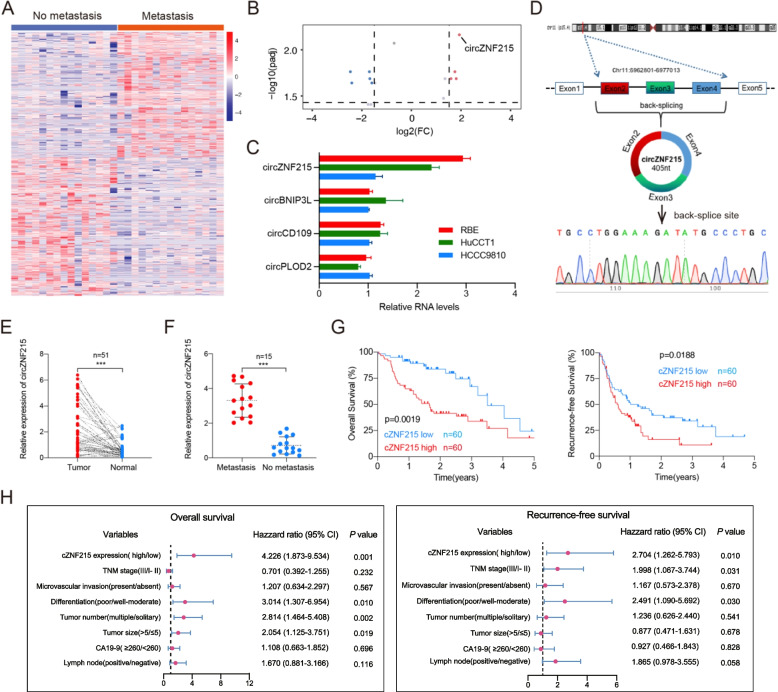
High expression of cZNF215 is correlated with iCCA metastasis and poor patient prognosis. **A** Clustered heat map of all differentially expressed circRNAs in 15 iCCA tissues with and without extrahepatic metastasis, respectively. **B** Volcano plot compared the expression fold changes of circRNAs for iCCA tissues with extrahepatic metastases versus without extrahepatic metastases. **C** Quantitative real-time PCR analysis showed that the levels of cZNF215 expression were the highest among the 4 upregulated circRNAs in RBE, HuCCT1, and HCCC9810 cell lines. **D** Schematic illustration of cZNF215 locus with specific primers and Sanger sequencing result of cZNF215. **E** qRT-PCR to detect the expression of cZNF215 in 51 iCCA tissues and paired normal tissues. **F** Relative RNA levels of cZNF215 in iCCA tissues (*n=*15) with and without extrahepatic metastasis, respectively. **G** Kaplan-Meier analysis showing the association of cZNF215 expression with OS or RFS in iCCA tissues. **H** Multivariate analysis of OS and RFS prognostic indicators. Data represent means±SD of at least three independent experiments

To determine whether circRNAs contribute to iCCA metastasis, we analyzed the differences in circRNA expression profiles using circRNA-seq from 15 primary iCCA tissues with or without postoperative metastases after surgery (Fig. 1A). Of the identified circRNAs, there were 4 significantly upregulated circRNAs (Fig. 1B). To further screen out the target circRNA, we conducted qRT-PCR to measure the levels of the 4 upregulated circRNAs in the iCCA cell lines (HuCCT1, RBE, and HCCC9810). The analysis showed that, compared with the other 3 circRNAs, the expression of cZNF215 was dramatically enhanced in RBE and HuCCT1 cell lines (Fig. 1C). As shown in Fig. 1B-C, cZNF215 expression showed the most significant difference, and its expression levels were the highest among the 4 upregulated circRNAs according to the RNA-seq data and qRT-PCR results. Accordingly, we chose cZNF215 for further study.

To identify the circular characteristics of cZNF215, Sanger sequencing was performed to identify the back-splice junctions (Fig. 1D). cZNF215 is derived from exons 2, 3 and 4 of the *ZNF215* gene, as well as transcript variant 1 (transcript ID: ENST00000278319.10, transcript name: ZNF215-201). ZNF215-201 is the only transcript variant that can be amplified by RT-PCR using the mRNA primers. Moreover, we found that cZNF215 was resistant to RNase R treatment, whereas the linear RNAs were degraded by RNase R, indicating that cZNF215 was circular, not linear (Supporting Fig. S1A). Additionally, oligo(dT)18 and random hexamer primers were applied to validate the circular feature of cZNF215, and cZNF215 was decreased in reverse-transcription efficiency by oligo(dT)18 primers due to the lack of a poly-A tail (Supporting Fig. S1B). In the actinomycin D assay, cZNF215 had a longer half-life than mZNF215 due to its circular structure (Supporting Fig. S1C).

Finally, subcellular RNA fractionation assays and fluorescent in situ hybridization (FISH) assays showed that cZNF215 was located primarily in the cytoplasm, suggesting that cZNF215 may exert its biological function there (Supporting Fig. S1D-E).

To explore the association between cZNF215 expression and clinicopathological features, the levels of cZNF215 were measured by qRT‒PCR in iCCA tissues with or without metastases (*n=*15), as well as in paired iCCA and adjacent normal tissue samples (*n=*51). cZNF215 expression was considerably upregulated in iCCA tissues compared with normal tissues, and the same results were found in iCCA tissues with metastases and without metastases, suggesting that cZNF215 might be correlated with poor prognosis and metastasis in iCCA patients (Fig. 1E-F). In addition, the expression of cZNF215 in 120 iCCA tissues (including the previous 30 samples) was examined. The patients were divided into two groups (high cZNF215 expression group (*n=*60) and low cZNF215 expression group (*n=*60)) according to the median expression of cZNF215. The results showed that the higher cZNF215 expression group had shorter overall survival (OS) and recurrence-free survival (RFS) (Fig. 1G). Consistently, we found that higher cZNF215 expression was correlated with tumor number, tumor size, lymph node metastasis, microvascular invasion (MVI) and advanced TNM tumor stage, showing that cZNF215 was correlated with the growth and metastasis of iCCA (Table 1). Next, univariate and multivariate analyses were conducted to assess the risk factors for OS and RFS. The results of univariate analysis showed that tumor number, tumor differentiation, lymph node status, serum CA19-9 level, TNM stage, tumor size, and cZNF215 expression were correlated with OS and RFS (Supporting Table S1). In multivariate regression analysis, multiple tumor number, poorer tumor differentiation, larger tumor size, and high cZNF215 expression were independent risk factors for OS, while the poorer tumor differentiation, advanced TNM stage and higher cZNF215 expression were regarded as independent risk factors for RFS (Corrected Fig.1H, corrected Table S2). Collectively, high cZNF215 expression predicted poor prognosis, suggesting that cZNF215 may be associated with iCCA metastasis and progression.

**Table 1:** Clinical characteristics of 120 iCCA patients based on cZNF215 expression levels

**Variables**	**Low cZNF215** **(*n* = 60)**	**High cZNF215** **(*n* = 60)**	***P* value**
Age, year, >60/≤60	27/33	26/34	0.854
Gender, male/female	28/32	32/28	0.465
Ascites, present/absent	3/57	10/50	0.075
Hepatolithiasis, present/absent	1/59	1/59	0.999
CA19-9, ≥260/<260	20/40	22/38	0.702
Tumor size (cm) >5/≤5	34/26	45/15	**0.034**
Tumor number, multiple/solitary	13/47	23/37	**0.046**
Differentiation, poor/well-moderate	44/16	46/14	0.673
MVI, present/absent	7/53	19/41	**0.008**
Lymph node, positive/negative	8/52	17/43	**0.043**
Cirrhosis, with/without	5/55	8/52	0.378
TNM stage, III/I- II	29/31	43/17	**0.009**
Chronic viral hepatitis B, present/absent	14/46	9/51	0.3538

*iCCA* Intrahepatic cholangiocarcinoma, *MVI* Microvascular invasion, *TNM* Tumor-node-metastasis

### cZNF215 facilitates iCCA progression and metastasis in vitro and in vivo

**Fig. 2:**
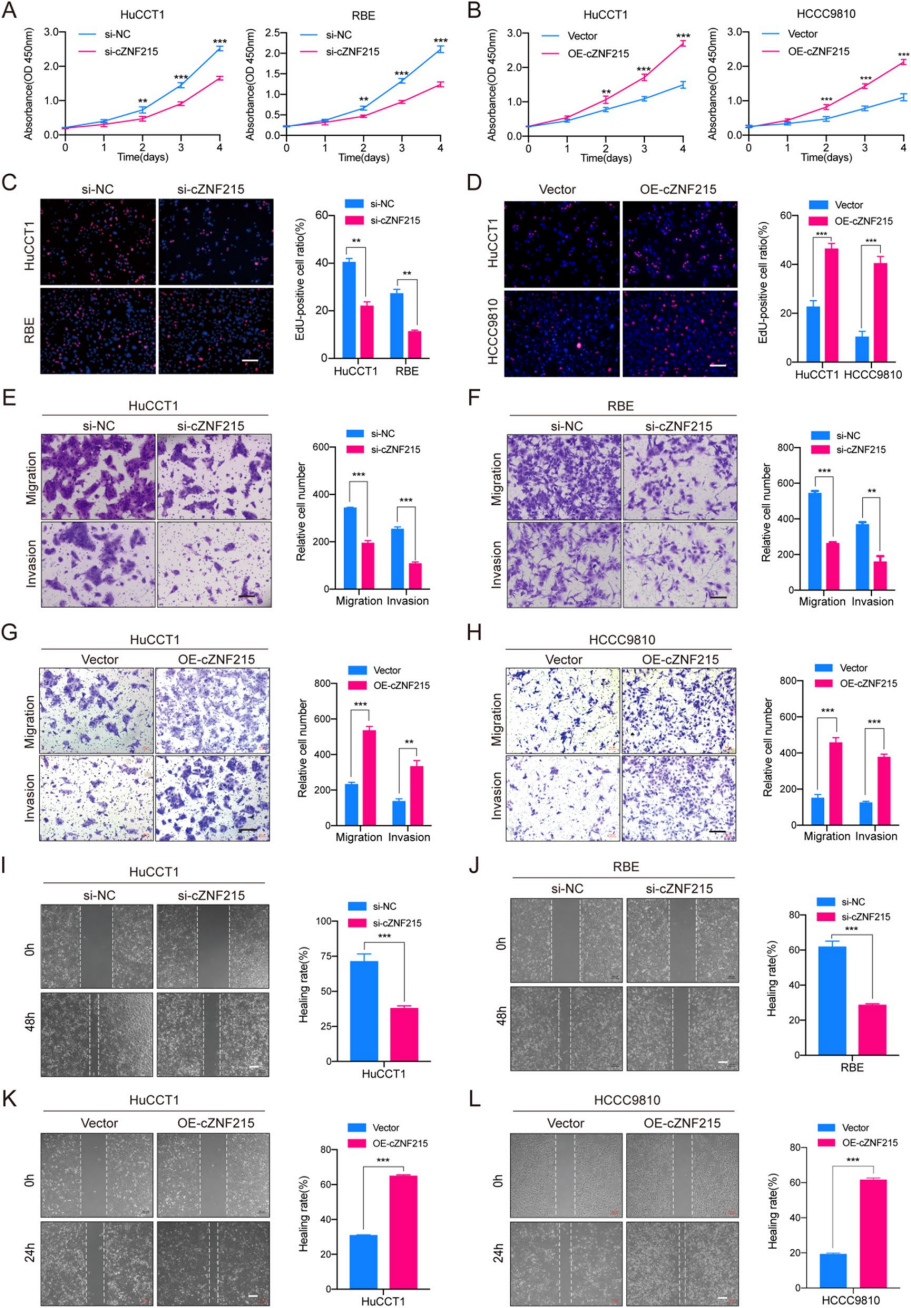
cZNF215 facilitates cell proliferation, migration and invasion of iCCA cells in vitro. **A** and **B** Cell Counting Kit-8 (CCK-8) assays were performed to examine the role of cZNF215 in cell proliferation of iCCA cells upon knockdown and overexpression of cZNF215. **C** and **D** EdU cell proliferation assays of indicated iCCA cells. **E**-**H** Transwell migration and matrigel invasion assays of indicated iCCA cells. **I**-**L** Wound healing assays of indicated iCCA cells. Abbreviations: NC, normal control; OE, overexpression. Data represent means±SD of at least three independent experiments. Scale bars, 200 μm

To evaluate the role of cZNF215 in iCCA progression and metastasis, small interfering RNA (siRNA) was used to silence cZNF215 in RBE and HuCCT1 cells (Supporting Fig. S2A-B), and a lentivirus expression vector was applied to overexpress cZNF215 in HuCCT1 and HCCC9810 cells (Supporting Fig. S2C-D). Knockdown of cZNF215 significantly suppressed cell proliferation, cell cycle progression, colony formation, cell migration and invasion of the RBE and HuCCT1 cells, while we observed the opposite results after cZNF215 was overexpressed (Fig. 2A-L, Supporting Fig. S2E-H).

**Fig. 3:**
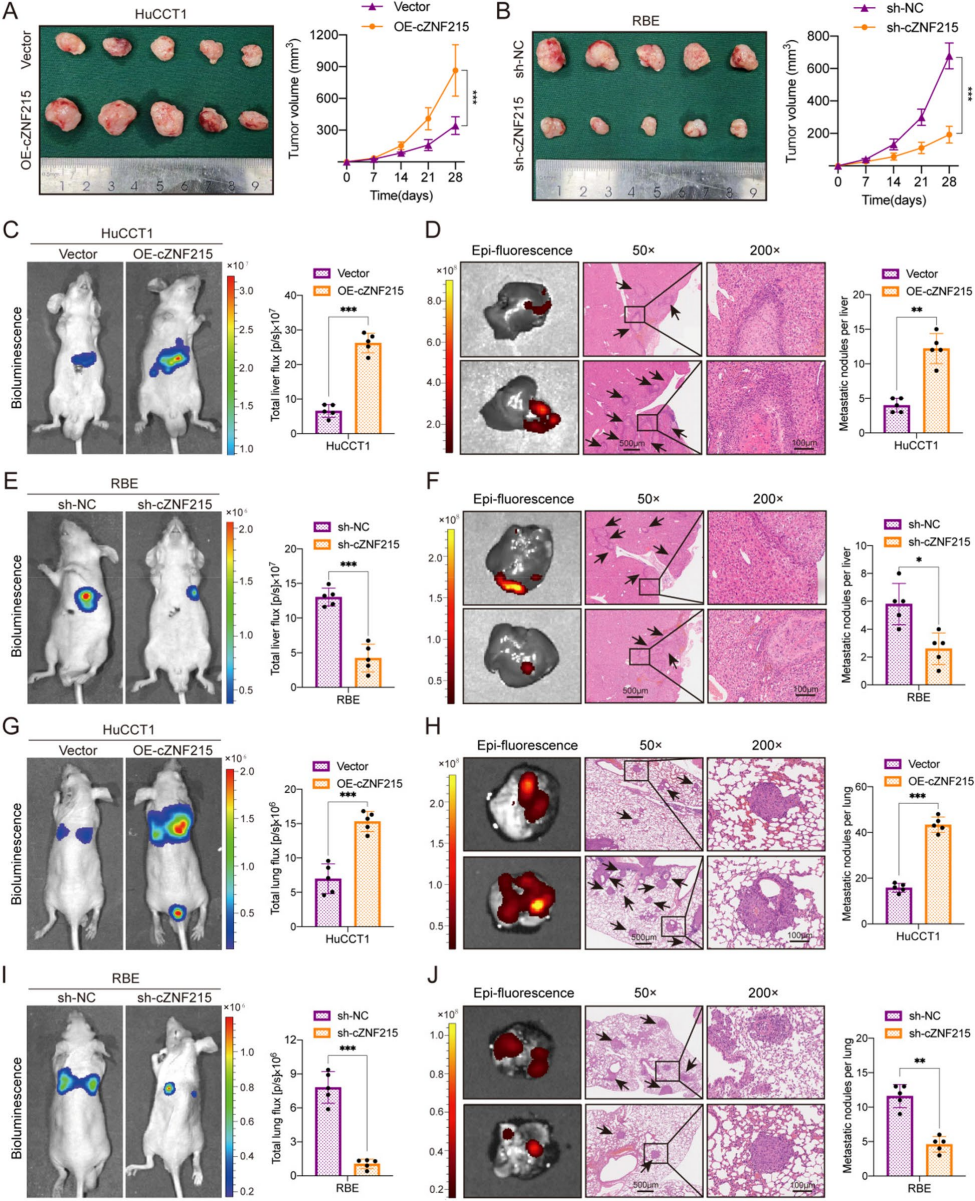
cZNF215 facilitates iCCA progression and metastasis in vivo. **A** and **B** Subcutaneous xenografts of the indicated cells dissected from nude mice, and growth curves of tumor volumes were calculated in the subcutaneous xenograft models (*n=*5). **C** and **E** Representative bioluminescent images after inoculation with HuCCT1 and RBE cells, respectively, in liver orthotopic-implantation models (*n=*5). **D** and **F** Representative fluorescent images and hematoxylin-eosin (HE) staining of metastatic nodules in liver orthotopic-implantation models (*n=*5). **G** and **I** Representative bioluminescent images after inoculation with HuCCT1 and RBE cells, respectively, in lung metastasis models (*n=*5). **H** and **J** Representative fluorescent images and HE staining of metastatic nodules in lung metastasis models (*n=*5)

To further explore the biological function of cZNF215 in vivo, stable iCCA cell lines with gain of function and loss of function of cZNF215 were established. cZNF215 was overexpressed in HuCCT1 cells by lentiviral transfection and knocked down through short hairpin RNA (shRNA) in RBE cells, which were labeled with firefly luciferase allowing for tracking by the in vivo imaging system (IVIS). As a result, tumor growth was markedly promoted by cZNF215 overexpression and suppressed after cZNF215 knockdown in subcutaneous implantation nude mouse models (Fig. 3A-B and Supporting Fig. S3A-B). In liver orthotopic implantation and lung metastasis models, the IVIS data indicated that overexpression of cZNF215 resulted in a larger tumor volume and more metastatic foci in both the liver and lung. Conversely, loss of cZNF215 suppressed iCCA metastasis in both models (Fig. 3C-J). Taken together, these data demonstrated that high cZNF215 expression facilitated iCCA growth and metastasis in vitro and in vivo.

### cZNF215 inactivates the PTEN/AKT pathway by promoting PTEN oxidation

To elucidate the detailed mechanisms of cZNF215 in facilitating iCCA progression and metastasis, RNA-seq was conducted to investigate the potential genes regulated by cZNF215 overexpression. The results of RNA-seq showed that 344 genes (159 upregulated and 185 downregulated) were differentially expressed (Supporting Fig. S4A). Moreover, we found that the PI3K-AKT signaling pathway was one of the most significantly enriched pathways through Kyoto Encyclopedia of Genes and Genomes (KEGG) pathway analysis (Supporting Fig. S4B). In addition, Gene Ontology (GO) analysis revealed that cZNF215 could modulate some essential biological processes, including cell proliferation, cell adhesion, and cell migration (Supporting Fig. S4C).

**Fig. 4:**
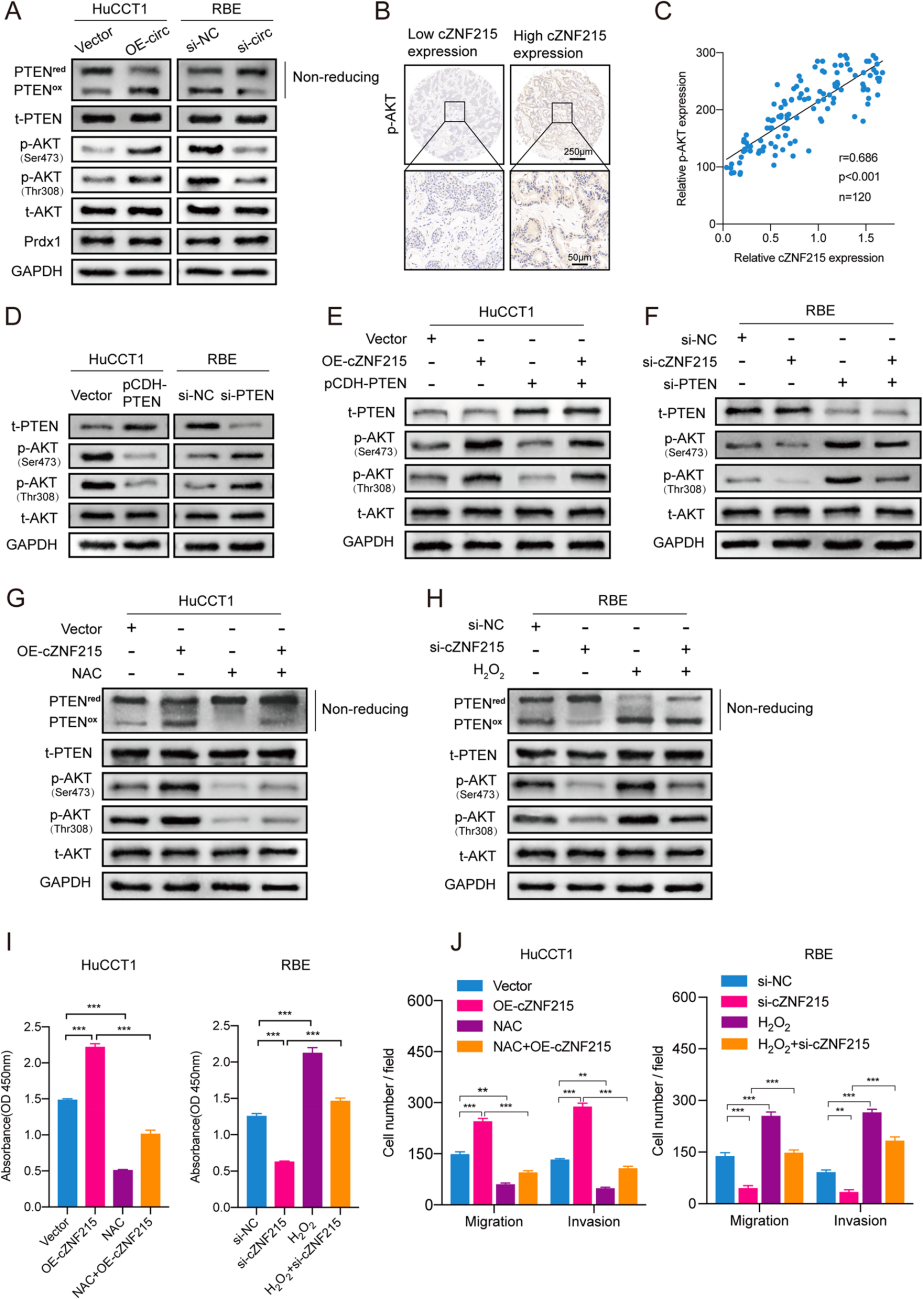
cZNF215 inactivates the PTEN/AKT pathway by promoting PTEN oxidation. **A** Western blot assays were conducted to analyze the expression of some relatively crucial proteins of PTEN/AKT pathway in both HuCCT1 and RBE cells. **B** Representative immunohistochemical (IHC) staining of p-AKT^Ser473/Thr308^ of iCCA tissues with high cZNF215 expression and low cZNF215 expression. **C** The association between p-AKT^Ser473/Thr308^ and cZNF215 levels of 120 iCCA tissues. **D** Western blot analysis showing the expression of t-PTEN, p-AKT^Ser473/Thr308^ and t-AKT in HuCCT1 cells transfected with vectors expressing PTEN or in RBE cells transfected with PTEN siRNA. **E** and **F** Western blot assays were performed to detect the expression of t-PTEN, p-AKT^Ser473/Thr308^ and t-AKT in HuCCT1 cells cotransfected with indicated vectors and RBE cells cotransfected with indicated siRNAs. **G** and **H** Western blot assays were conducted to examine the expression of PTEN^red^, PTEN^ox^, t-PTEN, p-AKT^Ser473/Thr308^ and t-AKT in HuCCT1 cells transfected with cZNF215 vectors and treated with NAC, or RBE cells transfected with cZNF215 siRNAs and treated with H_2_O_2_. **I** CCK8 assays showing the proliferation ability of iCCA cells among different groups. **J** Transwell assays analysis showing the migration and invasion capacity in iCCA cells. Abbreviations: PTEN^red^, reduced PTEN; PTEN^ox^, oxidized PTEN; t-PTEN, total PTEN; p-AKT^Ser473/Thr308^, phosphorylation of AKT on Ser473 and Thr308; t-AKT, total AKT; NAC, N-acetylcysteine

*PTEN* has been validated as a tumor suppressor. PI3K/AKT, a canonical oncogenic signaling pathway, is often abnormally activated in diverse cancers, and inactivation of PTEN can activate this pathway [29]. Furthermore, PTEN activity is negatively regulated through oxidation. Consequently, we speculated that cZNF215 facilitated iCCA growth and metastasis through oxidation-induced inactivation of the PTEN/AKT pathway. As the western blot results showed, overexpression of cZNF215 in HuCCT1 cells indeed enhanced PTEN oxidation (PTEN^ox^) and AKT phosphorylation on Ser473 (p-AKT^Ser473^) and Thr308 (p-AKT^Thr308^) in the nonreducing state, while knockdown of cZNF215 in RBE cells reduced the levels of PTEN^ox^ and p-AKT^Ser473/Thr308^ (Fig. 4A). To confirm this observation, we examined the correlation between cZNF215 and p-AKT in iCCA tissues. Noticeably, immunohistochemical (IHC) staining of p-AKT^Ser473/Thr308^ in iCCA tissues showed that, compared with the low cZNF215 expression group, the high cZNF215 expression group had stronger signal intensity (Fig. 4B). Consistently, cZNF215 expression was positively associated with the IHC score of p-AKT^Ser473/Thr308^ in 120 iCCA samples (Fig. 4C).

We then explored whether cZNF215-induced progression and metastasis of iCCA was mediated by inactivation of PTEN, which consequently led to AKT phosphorylation. Western blot analysis indicated that the levels of AKT^Ser473/Thr308^ phosphorylation were dramatically decreased by overexpression of PTEN (pCDH-PTEN) and increased upon PTEN knockdown (Fig. 4D). As expected, overexpression of PTEN partially alleviated the enhanced levels of AKT^Ser473/Thr308^ phosphorylation caused by cZNF215 overexpression in HuCCT1 cells. In contrast, knockdown of PTEN restored the decreased levels of AKT^Ser473/Thr308^ phosphorylation induced by silencing cZNF215 in RBE cells (Fig. 4E-F).

To further investigate whether cZNF215 promotes PTEN oxidation and AKT phosphorylation, we treated HuCCT1 cells and RBE cells with N-acetylcysteine (NAC, a thiol-containing antioxidant that modulates the intracellular redox state) [30] and H_2_O_2_, respectively. Our results suggested that treatment of HuCCT1 with NAC decreased the levels of PTEN^ox^ and AKT^Ser473/Thr308^ phosphorylation and significantly reversed the enhanced levels of PTEN^ox^ and AKT^Ser473/Thr308^ phosphorylation caused by cZNF215 overexpression (Fig. 4G). On the other hand, treatment of RBE cells with H_2_O_2_ showed the opposite results (Fig. 4H). These data demonstrated that upregulation of cZNF215 promoted oxidation-induced inactivation of PTEN and AKT^Ser473/Thr308^ phosphorylation.

Functionally, CCK-8 and transwell assays were performed to validate that cZNF215 facilitated the proliferation, migration and invasion of iCCA cells, which could be abolished by NAC. Conversely, suppression of cell proliferation, migration and invasion of iCCA cells by knockdown of cZNF215 could be rescued by oxidation-induced inactivation of PTEN (Fig. 4I-J, Supporting Fig. S4D-E). To further verify the above results, SF1670, a highly potent and specific inhibitor of PTEN, was used to selectively block PTEN [31–33]. The concentration-dependent inhibition of PTEN was shown in RBE cells treated with SF1670 (Supporting Fig. S4F). Knockdown of cZNF215 suppressed phosphorylation of AKT^Ser473/Thr308^ and the cell proliferation, migration and invasion of iCCA cells, which was restored by SF1670 (Supporting Fig. S5G-J). Taken together, our data strongly demonstrated that overexpression of cZNF215 promoted iCCA growth and metastasis by oxidation-induced inactivation of the PTEN/AKT pathway.

### PRDX1 is involved in cZNF215-mediated PTEN/AKT pathway inactivation by interacting with cZNF215

**Fig. 5:**
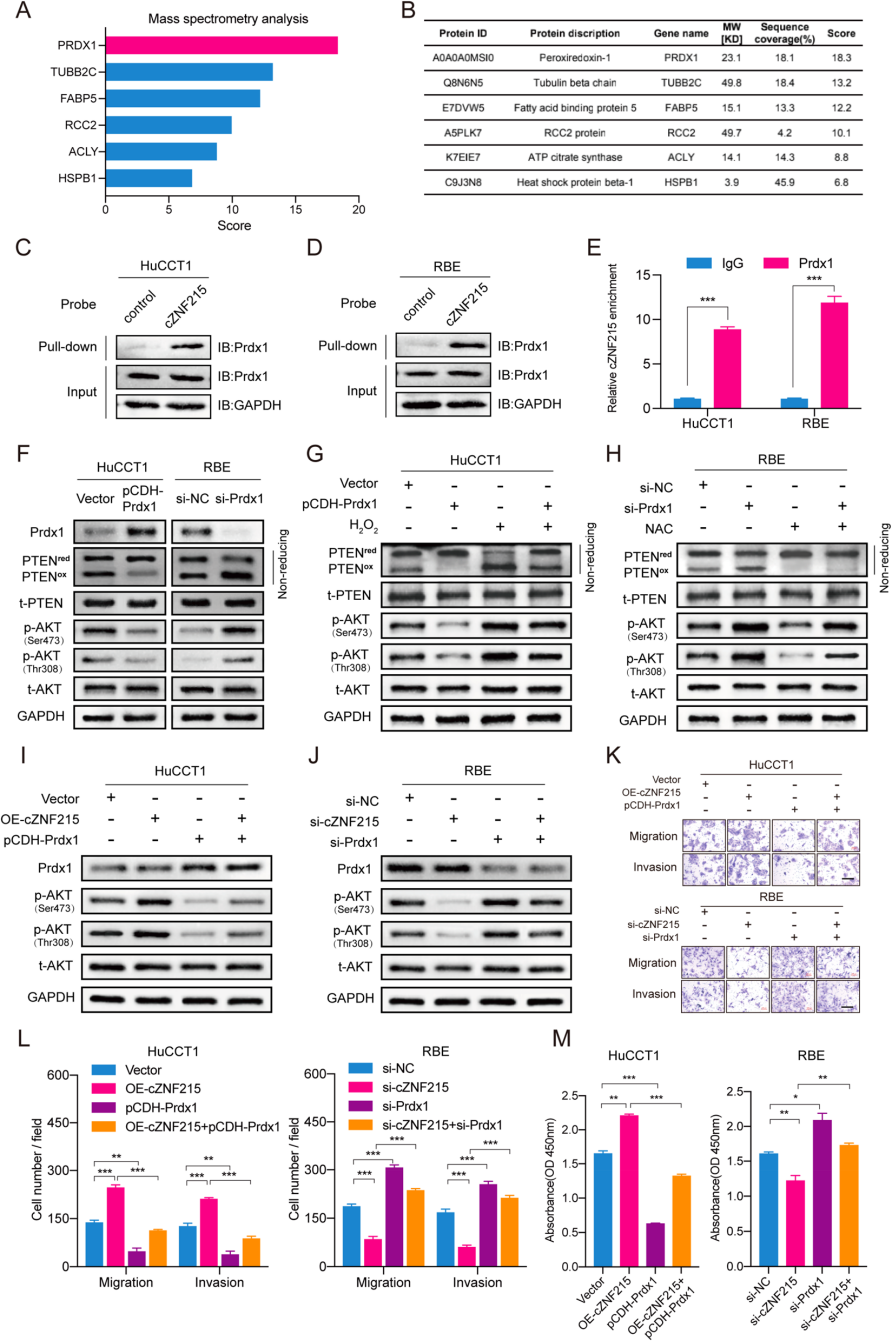
PRDX1 is involved in cZNF215-mediated PTEN/AKT pathway inactivation by interacting with cZNF215. **A** RNA pull-down assays were performed using HuCCT1 cells lysates, following by mass spectrometry (MS) analysis. **B** MS analysis showing PRDX1 is likely to be a cZNF215-binding protein. **C**, **D** and **E** RNA pull-down and RNA immunoprecipitation assays analysis determined the interaction between cZNF215 and PRDX1 in indicated cells. **F** Immunoblot assays analysis revealing the expression of PRDX1, PTEN^red^, PTEN^ox^, t-PTEN, p-AKT^Ser473/Thr308^ and t-AKT in HuCCT1 cells transfected with vectors expressing PRDX1 or RBE cells transfected with PRDX1 siRNA. **G** and **H** Immunoblot assays analysis indicating the expression of PRDX1, PTEN^red^, PTEN^ox^, t-PTEN, p-AKT^Ser473/Thr308^ and t-AKT in HuCCT1 cells transfected with vectors expressing PRDX1 and treated with H_2_O_2_, or RBE cells transfected with PRDX1 siRNA and treated with NAC. **I** and **J** Western blot for the expression of PRDX1, p-AKT^Ser473/Thr308^ and t-AKT in HuCCT1 cells cotransfected with indicated vectors or RBE cells cotransfected with indicated siRNAs. **K** and **L** Transwell assays analysis showing the migration and invasion capacity in iCCA cells. Scale bars, 200 μm. **M** CCK8 assays showing the proliferation ability of iCCA cells. Abbreviations: PTEN^red^, reduced PTEN; PTEN^ox^, oxidized PTEN; t-PTEN, total PTEN; p-AKT^Ser473/Thr308^, phosphorylation of AKT on Ser473 and Thr308; t-AKT, total AKT; NAC, N-acetylcysteine

To explore how cZNF215 modulated PTEN oxidation, which led to phosphorylation of AKT^Ser473/Thr308^ and tumor promotion, we first examined the protein-coding potential of cZNF215. We found that cZNF215 had little protein-coding potential due to the lack of an open reading frame (ORF) in circRNADb [34] (Supporting Fig. S6A). Next, we performed RNA immunoprecipitation (RIP) assays in HuCCT1 and RBE cells. The results showed that there was no significant difference in cZNF215 enrichment between the IgG group and the AGO2 group, suggesting that cZNF215 was unlikely to act as an “miRNA sponge” (Supporting Fig. S5B). In addition, cZNF215 was located primarily in the cytoplasm (Supporting Fig. S1D-E). Therefore, we hypothesized that cZNF215 might function as a protein scaffold. To validate this assumption, we conducted RNA pull-down assays by using a biotinylated cZNF215 probe in HuCCT1 cell lysates. Mass spectrometry (MS) analysis revealed that PRDX1, an antioxidant enzyme with a molecular weight of 23 KD, was the most likely RNA-binding protein for cZNF215 (Fig. 5A-B). Next, cZNF215 pull-down and RIP assays were performed to confirm the interaction between cZNF215 and PRDX1 in HuCCT1 and RBE cells, respectively (Fig. 5C-E).

PRDX1 was first reported as an antioxidant enzyme and was proven to be a versatile molecule modulating cell proliferation [24]. Many studies have revealed that PRDX1-regulated ROS-dependent signaling pathways play a key role in the development of various cancers, including breast cancer [35, 36], esophageal cancer [37, 38], lung cancer [39, 40], prostate cancer [41, 42], and pancreatic cancer [43, 44]. ROS are widely recognized as oncogenic factors that can be inhibited by PRDX1. Moreover, a study demonstrated that PRDX1 could promote the tumor inhibition effects of PTEN by binding PTEN and protecting it from H_2_O_2_-induced inactivation [28]. To verify this, we knocked down PRDX1 in RBE cells and overexpressed PRDX1 in HuCCT1 cells. Western blot analysis revealed that overexpression of PRDX1 considerably decreased the levels of PTEN^ox^ and AKT^Ser473/Thr308^ phosphorylation. In contrast, silencing PRDX1 showed the opposite effects (Fig. 5F). As a result, treatment of HuCCT1 cells with H_2_O_2_ dramatically enhanced the levels of PTEN^ox^ and AKT^Ser473/Thr308^ phosphorylation and could restore the decreased levels of PTEN^ox^ and AKT^Ser473/Thr308^ phosphorylation caused by PRDX1 overexpression. Conversely, treatment of RBE cells with NAC had the opposite effects (Fig. 5G-H). These data demonstrated that PRDX1 could indeed protect PTEN from oxidation-induced inactivation, thereby inhibiting AKT^Ser473/Thr308^ phosphorylation. Functionally, we conducted CCK-8 and transwell assays to confirm that suppression of cell proliferation, migration and invasion by PRDX1 overexpression could be abolished by H_2_O_2_ (Supporting Fig. S5C-F).

PRDX1 is a safeguard for the activity of PTEN and is essential for the tumor suppressive functions of PTEN [28]. Accordingly, we reasoned that cZNF215 might regulate PTEN oxidation by interacting with PRDX1 in iCCA cells. The immunoblotting results showed that, as expected, overexpression of PRDX1 attenuated the promoting effect of cZNF215 overexpression on AKT^Ser473/Thr308^ phosphorylation, whereas silencing of PRDX1 rescued the cZNF215-induced reduction in AKT^Ser473/Thr308^ phosphorylation (Fig. 5I-J). Functionally, overexpression of PRDX1 remarkably attenuated cell proliferation, migration and invasion that were enhanced by cZNF215 overexpression. In contrast, silencing PRDX1 eliminated the inhibitory effects of cZNF215 knockdown on cell proliferation, migration and invasion (Fig. 5K-M). Collectively, these data demonstrated that cZNF215 facilitated iCCA progression and metastasis by promoting oxidation-induced inactivation of PTEN by interacting with PRDX1.

### cZNF215 facilitates PTEN oxidation by preventing the interaction between PRDX1 and PTEN

**Fig. 6:**
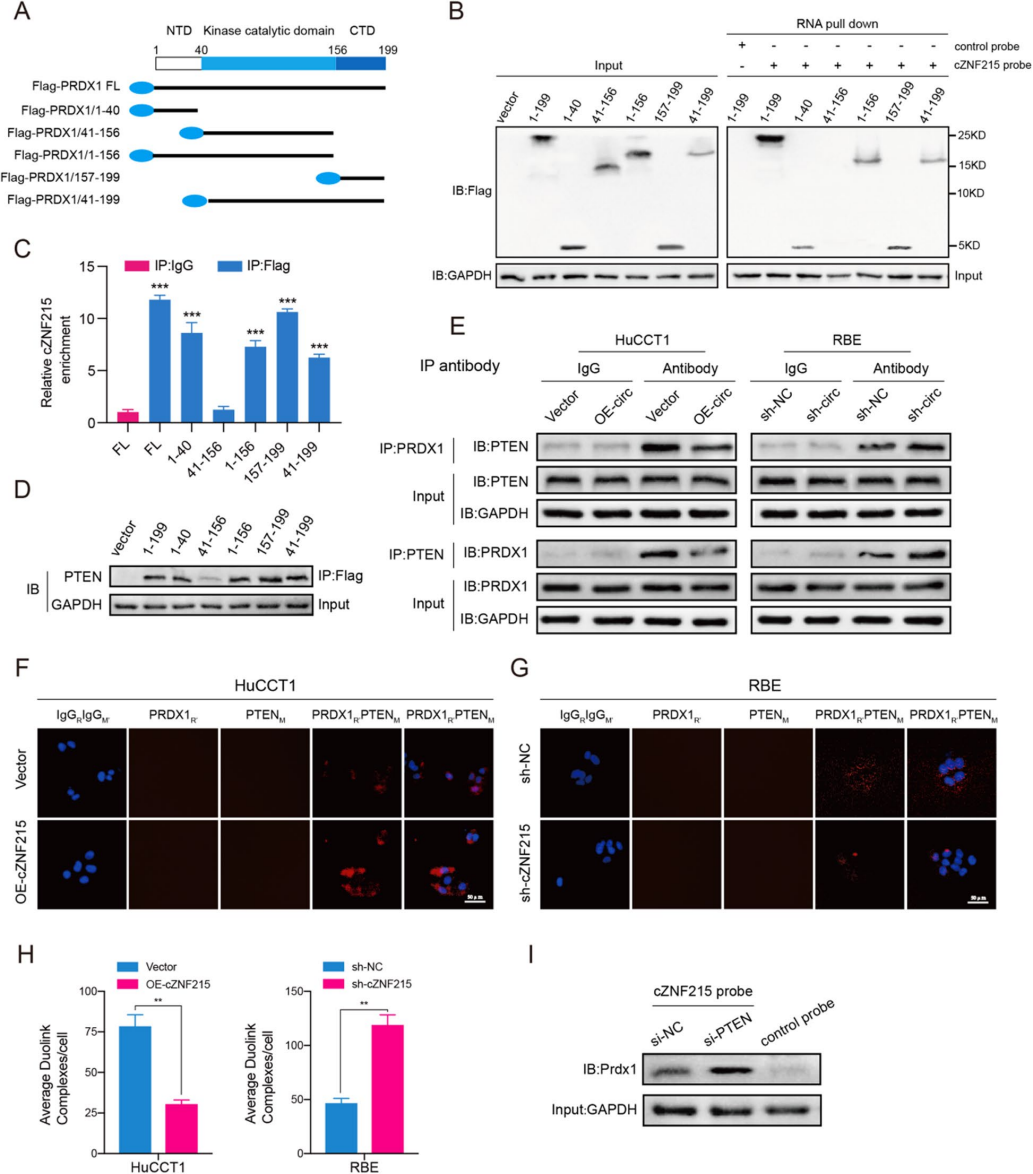
cZNF215 facilitates PTEN oxidation by blocking the interaction between PRDX1 and PTEN. **A** The diagrams of PRDX1 domain structure and Flag-tagged PRDX1 truncations. **B** Immunoblot assays were performed to detect the expression of PRDX1 truncations and full length of the lysates from HEK293T cells transfected with the indicated vectors (left); RNA pull-down and Immunoblot assays analysis showing the proteins enriched with cZNF215 (right). **C** RIP assays analysis showing the results from cZNF215 pull-down. **D** The binding domain of PRDX1 responsible for its interaction with PTEN was validated by Co-IP assays in HEK293T cells. **E** The interaction between PRDX1 and PTEN was confirmed by Co-IP assays in the HuCCT1 cells transfected with indicated vectors or RBE cells transfected with indicated shRNA. **F** and **G** The interaction between PRDX1 and PTEN was further verified by Duolink in situ proximity ligation assay (PLA) assay in the above cells. Scale bars, 50 μm. **H** The statistical analysis results of average Duolink complexes per cell of PLA assay. **I** RNA pull-down and immunoblot assays were conducted to detect the cZNF215-PRDX1 complex in HuCCT1 cells transfected with the PTEN siRNA

As shown in Fig. 4A and 5I-J, there were no obvious changes in PRDX1 levels after regulation of cZNF215. Hence, we presumed that cZNF215 could affect the interaction between PRDX1 and PTEN. To determine the interaction between PRDX1 and cZNF215, we conducted RNA pull-down and RIP assays using a series of Flag-tagged PRDX1 truncation mutants (Fig. 6A). The results showed that the N-terminal domain (NTD) of PRDX1 (1-40 aa) and the C-terminal domain (CTD) of PRDX1 (157-199 aa) were critical for its interaction with cZNF215 (Fig. 6B-C). Noticeably, a previous study revealed that PTEN interacted with the NTD of PRDX1 (1-40 aa) and the CTD of PRDX1 (157-199 aa) [28]. Consistently, our results of the Co-IP assay also demonstrated that PTEN primarily binds to the NTD and CTD of PRDX1 (Fig. 6D). In summary, these data demonstrated that cZNF215 and PTEN have the same PRDX1-binding domain, suggesting that cZNF215 and PTEN are likely to competitively bind to PRDX1.

Next, we performed Co-IP assays to determine whether cZNF215 could affect the interaction between PRDX1 and PTEN. As expected, overexpression of cZNF215 in HuCCT1 cells significantly weakened the PRDX1-PTEN interaction. In contrast, the interaction between PRDX1 and PTEN was strengthened when cZNF215 was silenced (Fig. 6E). Additionally, the results were also confirmed by duolink in situ proximity ligation assay (PLA) (Fig. 6F-H). We next performed cZNF215 pull-down assays and showed that more PRDX1 was enriched by cZNF215 upon knockdown of PTEN compared with the control group, which further validated the competitive binding of cZNF215 and PTEN to PRDX1 (Fig. 6I). In conclusion, our data demonstrated that cZNF215 blocked the interaction between PRDX1 and PTEN by competitively binding to PRDX1, thus resulting in oxidation-induced inactivation of PTEN and AKT phosphorylation and finally facilitating iCCA progression and metastasis.

### cZNF215 is a potential therapeutic target and enhances the antitumor effects of ipatasertib in vivo

Ipatasertib is a specific inhibitor of the serine/threonine protein kinase AKT with potential antineoplastic activity and has been widely used in various studies [45–47]. On the other hand, our previous data demonstrated that knockdown of cZNF215 inhibits the PI3K/AKT pathway by enhancing the activation of PTEN. Based on the fact that knockdown of cZNF215 inhibits the PI3K/AKT pathway in a different manner compared with the AKT inhibitor ipatasertib, we wondered whether it was possible for cZNF215 to strengthen the effect of ipatasertib. To verify this hypothesis, a CCK-8 assay was conducted to examine the therapeutic potential of targeting cZNF215 in combination with ipatasertib in iCCA cells. The findings showed that cZNF215 knockdown markedly reduced the IC50 values of ipatasertib, suggesting that cZNF215 knockdown could boost the antitumor effect of ipatasertib (Supporting Fig. S6A-B).

**Fig. 7:**
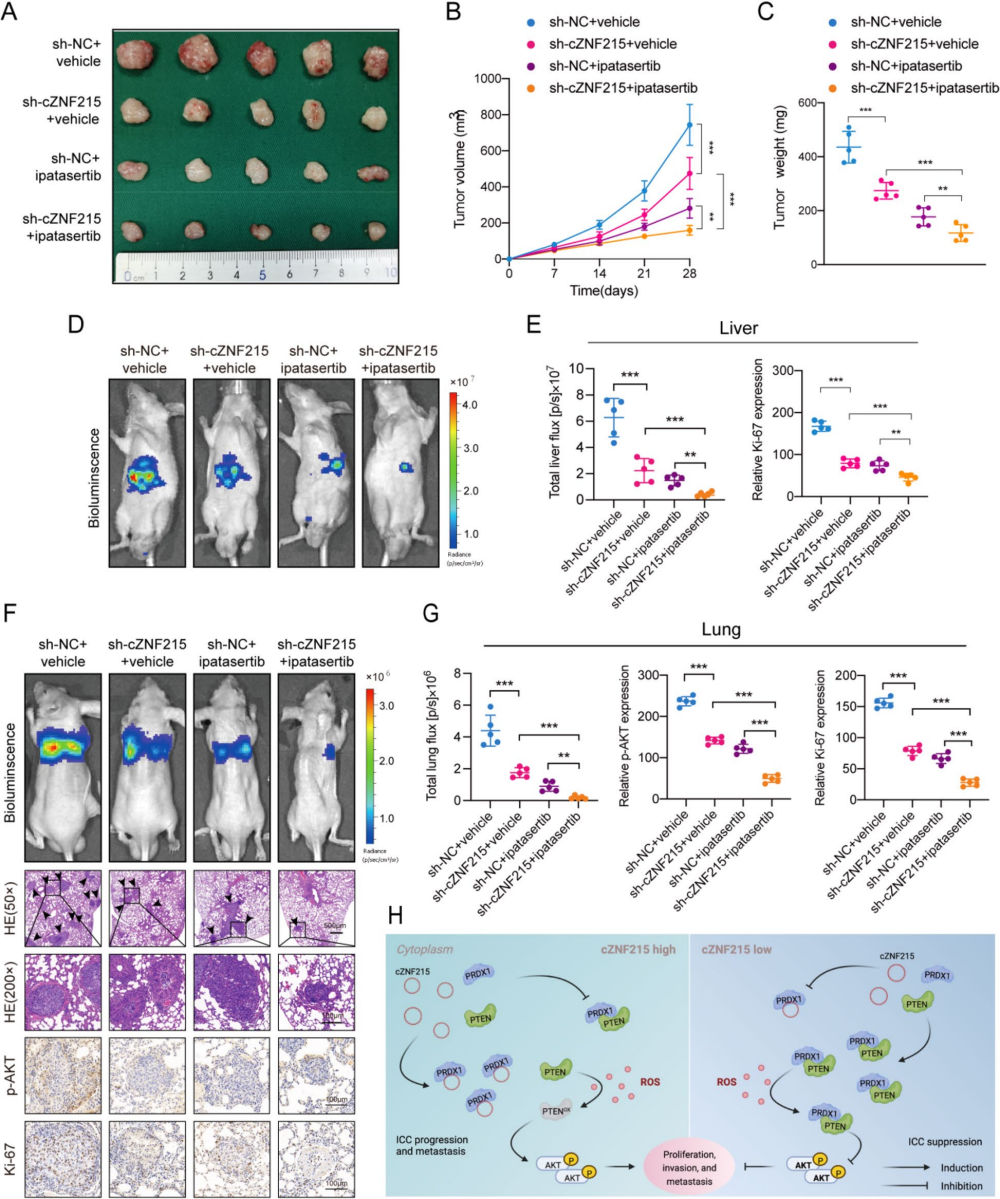
Knockdown of cZNF215 boosts anti-tumor effect of ipatasertib in vivo. **A** RBE cells (stably expressing sh-NC vector or sh-cZNF215) were used to establish subcutaneous xenograft models. The figure showed representative images of excised tumors at the end of the experiment. **B** and **C** Tumor volume and tumor weight of subcutaneous xenografts. **D** and **E** Representative bioluminescent images of liver orthotopic-implantation models, and quantitative analysis of bioluminescent results and Ki-67 staining results. **F** Representative fluorescent images of lung metastatic nodules (top); representative HE-stained images of lung metastatic nodules and IHC staining of p-AKT^Ser473/Thr308^ and Ki-67 in lung metastases (bottom). **G** Quantitative analysis of bioluminescent results, and staining results of p-AKT^Ser473/Thr308^ and Ki-67. **H** Summary figure of the mechanism that how cZNF215 facilitates iCCA growth and metastasis

To further confirm the above results, we assessed the effect of cZNF215 on the progression and metastasis of iCCA cells treated with ipatasertib in vivo. As expected, in subcutaneous implantation nude mouse models, tumor growth was notably suppressed by cZNF215 knockdown or ipatasertib administration alone, and this suppressive effect was further strengthened by the combination of cZNF215 knockdown with ipatasertib treatment (Fig. 7A-C). Subsequent IHC staining of xenografts also validated that silencing cZNF215 combined with ipatasertib treatment led to the most obvious inhibition of p-AKT^Ser473/Thr308^ and the weakest Ki-67 staining among the four groups (Supporting Fig. S7A-B). Additionally, similar results were obtained in both liver orthotopic-implantation models and lung metastasis models. Furthermore, silencing of cZNF215 in RBE cells markedly strengthened ipatasertib-mediated iCCA inhibition, as indicated by fewer metastatic foci compared with ipatasertib treatment alone (Fig. 7D-G and Supporting Fig. S7C). In summary, our data demonstrated that targeting cZNF215 to heighten the antitumor effect of ipatasertib might be a potential therapeutic strategy for iCCA.

## Discussion

ICCA is an aggressive and heterogeneous malignancy resulting from a complicated multifactorial carcinogenesis process, and it is characterized by late diagnosis and a poor prognosis. With the widespread application of bioinformatics technologies and high-throughput sequencing, many circRNAs have been identified at a phenomenal rate, but the function and modulation of most circRNAs remain unknown. Although the aberrant expression of circRNAs is correlated with the development and metastasis of iCCA, the biological functions of only a few circRNAs have been studied. For instance, upregulation of circNFIB suppressed iCCA progression and metastasis and could delay trametinib resistance in vitro and in vivo [48]. In another study, circHMGCS1-016 was found to be upregulated in iCCA tissues and to induce iCCA cell proliferation, invasion, and metastasis [49]. Moreover, high expression of circCCAC1 was positively associated with tumor growth, vascular invasion, poorer prognosis and recurrence. In vivo studies further showed that increased circCCAC1 levels in circulating cells accelerated both iCCA tumorigenesis and metastasis [50]. In this study, by performing circRNA-seq, we discovered that cZNF215 was dramatically upregulated in metastatic iCCA tissues, and it was confirmed as a tumor promoter. Moreover, the levels of cZNF215 were associated with lymph node metastasis, tumor size, tumor number, MVI stage, and advanced TNM tumor stage. Additionally, patients with high cZNF215 expression had a poor prognosis. Taken together, our data demonstrated that cZNF215 is closely related to the progression and metastasis of iCCA, and it can be used as a prognostic factor for iCCA patients.

The role of PTEN mainly relies on its lipid phosphatase activity by restraining the PI3K/AKT oncogenic pathway in the cytoplasm. Activation of the PI3K/AKT pathway is often accompanied by oxidation-induced inactivation of PTEN in the development of cancer [25–27]. ROS are widely considered to promote tumorigenesis via oxidation-induced inactivation of PTEN. Previous studies have revealed that PRDX1 restrains AKT-driven tumorigenesis by binding to PTEN to protect PTEN lipid phosphatase activity from oxidation-induced inactivation [24, 28, 51]. In this study, we showed that cZNF215 could promote oxidation-induced inactivation of PTEN by competitively interacting with PRDX1, which caused disassociation of PTEN and PRDX1 and finally contributed to oxidation of PTEN. Our study demonstrated that cZNF215 played a vital regulatory role via the PRDX1/PTEN/AKT axis in iCCA, which expands our understanding of the intracellular regulatory mechanism of the PI3K/AKT pathway.

To date, the biological functions of only a small fraction of the identified circRNAs have been revealed, and the vast majority of them are considered to act as miRNA sponges or protein sponges in the cytoplasm [52, 53]. The competing endogenous RNA (ceRNA) hypothesis is the most well-studied mechanism, but it is controversial. The analysis of many annotated circRNAs has revealed that most circRNAs do not have as many miRNA-binding sites as expected, and the level of target sites required for competing with miRNAs is likely to be exaggerated in some models for their role [54, 55]. Additionally, the functions of circRNAs may be more complex. Previous mechanistic studies have shown that circRNAs can modulate protein function through interactions with RNA-binding proteins (RBPs) [52]. In this study, we found that the expression of cZNF215 was dramatically upregulated in iCCA tissues and cell lines. We first confirmed that cZNF215 was unlikely to function as an miRNA sponge. Next, we identified a potential mechanism by which cZNF215 facilitated oxidation-induced inactivation of PTEN and AKT^Ser473/Thr308^ phosphorylation by restraining the interaction between PRDX1 and PTEN. Mechanistically, we revealed that cZNF215 bound to the NTD and CTD region of PRDX1, which contained the domain responsible for the interaction between PRDX1 and PTEN. When cZNF215 bound PRDX1, it restrained the binding of PRDX1 to PTEN, resulting in oxidation-induced inactivation of PTEN due to loss of protection by PRDX1, finally leading to the promotion of iCCA growth and metastasis (Fig. 7H).

ICCA is usually diagnosed at an advanced stage, and the incidence rate of iCCA is increasing worldwide [5]. Almost all iCCA patients require chemotherapy, while targeted agents and immunotherapy are only effective in specific patient groups [56]. Current diagnostic methods and therapeutic strategies for iCCA are not satisfactory; thus, new therapeutic measures are urgently needed. The combination of gemcitabine and cisplatin has been used for many years to treat patients with advanced/metastatic iCCA, but unfortunately, the median survival is only approximately 1 year [57]. A phase 2 clinical trial showed that nab-paclitaxel combined with gemcitabine-cisplatin could prolong median progression-free survival and overall survival in patients with advanced iCCA compared with gemcitabine-cisplatin alone [58]. Another study showed that circSMARCA5 upregulation decreased cell proliferation in cisplatin-treated as well as gemcitabine-treated cells and decreased the inhibitory concentration by 50% (IC50) of cisplatin and gemcitabine [59]. Our study found that cZNF215 knockdown could produce synergistic effects on ipatasertib, which also suggested that iCCA cells with low levels of cZNF215 might delay ipatasertib resistance. Noticeably, nucleic acid drugs are expected to become the third largest type of drug after small molecules and monoclonal antibodies, but many RNA-targeted drugs cannot be delivered directly into human cancer cells, leading to the failure of clinical trials evaluating these drugs. RNA nanotechnology, a bottom-up self-assembly of RNA structure at the nanoscale, may play an important role in cell manipulation and delivery [60, 61]. Guo et al. used RNA nanotechnology to successfully target prostate cancer, breast cancer, and colon cancer in animal models with nanoparticles (extracellular vesicles loaded with therapeutic RNA) [62]. Hence, utilizing the stability characteristics of cZNF215 combined with RNA nanotechnology may have greater development potential and application value than other RNA interference technologies for a new generation of iCCA targeted drugs.

## Conclusions

In summary, our study characterized a novel circRNA, cZNF215, and uncovered a novel mechanism by which cZNF215 competitively interacts with PRDX1 to promote the dissociation between PRDX1 and PTEN, subsequently leading to inactivation of the PTEN/AKT pathway and facilitating iCCA growth and metastasis. Upregulation of cZNF215 is correlated with metastasis and unfavorable prognosis in iCCA patients. Accordingly, cZNF215 may have the potential to serve as a therapeutic target and prognostic biomarker in iCCA patients.

### Supplementary Information

**Additional file 1.** Supporting Materials and Methods.

**Additional file 2:** **Figure S1. **The characteristics and localization of cZNF215.** (A)** qRT-PCR analysis for the expression of cZNF215 and ZNF215 mRNA in HuCCT1 and RBE cells treated with RNase R. β-actin mRNA was used as a negative control. (B) qRT-PCR analysis for the cZNF215 and ZNF215 mRNA using the template cDNA reverse-transcribed by random hexamer and oligo (dT)18 primers. (C) qRT-PCR analysis for the expression of cZNF215 and ZNF215 mRNA in HuCCT1 and RBE cells treated with Actinomycin D at indicated time points. (D) Subcellular RNA fractionation assays were conducted to determine the subcellular localization of cZNF215 and ZNF215 mRNA in HuCCT1 and RBE cells. U3 and β-actin were used as nuclear and cytoplasmic internal reference, respectively. (E) The localization of cZNF215 was further examined by FISH assays. Scale bar, 50 μm. **Figure S2. **cZNF215 promotes cell proliferation of iCCA cells *in vitro*. (A) and (B) Relative RNA levels of cZNF215 and ZNF215 mRNA after knockdown of cZNF215 in RBE and HuCCT1 cells. (C) and (D) Relative RNA levels of cZNF215 and ZNF215 mRNA after overexpression of cZNF215 in HuCCT1 and HCCC9810 cells. (E) and (F) Cell cycle analyses were performed to evaluate the effects of cZNF215 on cell cycle of iCCA cells. (G) and (H) Colony formation assays were conducted to assess the effects of cZNF215 on cell proliferation of iCCA cells.** Figure S3. **cZNF215 facilitates tumor growth of iCCA *in vivo*. (A) and (B) Effects of cZNF215 overexpression or knockdown on tumor weight in subcutaneous xenograft models.** Figure S4. **cZNF215 promotes iCCA proliferation and metastasis through inactivation of PTEN/AKT pathway. (A) Heatmap of differentially expressed genes of HuCCT1 cells transfected with the control and cZNF215-overexpressing lentiviral vectors. (B) and (C) The results of KEGG and GO analyses showed the main signaling pathways and the biological process affected by cZNF215 overexpression. (D) and (E) Transwell migration and matrigel invasion assays were performed in HuCCT1 cells treated with indicated vectors and NAC (an antioxidant), or RBE cells treated with cZNF215 siRNAs and H_2_O_2_. (F) Western blot analysis for p-AKT^Ser473/Thr308^ and t-AKT expression using protein extracts from RBE cells treated with SF1670 (a PTEN inhibitor) for 24h. (G) Western blot assay for PTEN, p-AKT^Ser473/Thr308^ and t-AKT protein levels in RBE cells transfected with cZNF215 siRNA or treated with SF1670 (0.4μM). GAPDH served as the internal reference. (H) Colony formation assays were conducted to examine colony formation capacity of RBE cells. (I) and (J) Transwell assays were performed to determine the migration and invasion capacity of RBE cells. Scale bars, 200 μm.** Figure S5. **PRDX1 is involved in cZNF215-mediated PTEN/AKT pathway inactivation by interacting with cZNF215. (A) Prediction of protein-coding potential of cZNF215 using the circRNADb algorithm. (B) RIP assays were conducted in HuCCT1 and RBE cells to determine the interaction between cZNF215 and AGO2. (C, D and E) Transwell migration and matrigel invasion assays were conducted in HuCCT1 cells treated with Prdx1 (an antioxidant enzyme) vectors and H_2_O_2_, or RBE cells treated with Prdx1 siRNAs and NAC. (F) CCK8 assays showed the proliferation ability of HuCCT1 and RBE cells. Scale bars, 200 μm.** Figure S6. **cZNF215 serves as a potential therapeutic target of iCCA. (A) and (B) Ipatasertib was used to incubate with HuCCT1 or RBE cells for 72h and the IC50 value was calculated by CCK-8 assays.** Figure S7.** Silencing ofcZNF215 enhances antitumor effect of ipatasertib* in vivo*. (A) and (B) Representative IHC staining of p-AKT^Ser473/Thr308^ and Ki-67, and quantitative analysis of p-AKT^Ser473/Thr308^ and Ki-67 staining results in xenografts. Scale bars, 100 μm. (C) Top,representative HE staining of metastatic foci in the livers. Scale bars, 200 μm. Bottom, representative IHC staining of Ki-67 in the livers. Scale bars, 100 μm.

**Additional file 3:** **Table S1.** Univariate analysisof several variables for OS and RFS. **Table S2.** Multivariate analysis ofseveral variables for OS and RFS. **Table S3.** The primers sequences wereas follows. **Table S4.** The target sequences of siRNAs were as follows. **Table S5.** Antibodies and reagents were as follows. **Table S6.** FISH probes used in this study.

## Data Availability

All data generated or analyzed during this study are included either in this article or in the supplementary information files. The raw data of circRNA-seq and RNA-seq have been deposited in the Genome Sequence Archive (GSA) in National Genomics Data Center, China National Center for Bioinformation/Beijing Institute of Genomics, Chinese Academy of Sciences.

## Abbreviations

circRNA: circular RNA
iCCA: intrahepatic cholangiocarcinoma
circRNA-seq: circRNA sequencing
RIP: RNA immunoprecipitation
FISH: fluorescence in situ hybridization
PRDX1: peroxiredoxin 1
PTEN: phosphatase and tensin homolog
ROS: Reactive oxygen species
NEM: N-ethylmaleimide
NAC: N-acetylcysteine
siRNA: small interfering RNA
shRNA: short hairpin RNA
ceRNA: competing endogenous RNA
RBPs: RNA-Binding Proteins
