# Correction: CircZNF215 promotes tumor growth and metastasis through inactivation of the PTEN/AKT pathway in intrahepatic cholangiocarcinoma

**DOI:** 10.1186/s13046-024-02977-1

**Published:** 2024-02-23

**Authors:** Wenwei Liao, Jinpeng Du, Lian Li, Xianquan Wu, Xing Chen, Qingbo Feng, Lin Xu, Xiangzheng Chen, Mingheng Liao, Jiwei Huang, Kefei Yuan, Yong Zeng

**Affiliations:** 1 Division of Liver Surgery, Department of General Surgery, West China Hospital, Sichuan University and Collaborative Innovation Center of Biotherapy, Chengdu, 610041 China; 2 Department of Hepatobiliary and Pancreatic Surgery, The First Afliated Hospital of Zhengzhou University, Zhengzhou, 450052 Henan China; 3 Department of General Surgery, The Fifth Afliated Hospital of Sun Yat-Sen University, Zhuhai, 519000 Guangdong China

**Correction** **:** **J Exp Clin Cancer Res 42, 125 (2023)**

**https://doi.org/10.1186/s13046-023-02699-w**

Following the publication of the original article [1], the authors identified errors in the body. Under the Results, the last paragraph of CircZNF215 is considerably increased in iCCA tissues, and high expression of cZNF215 is correlated with metastasis and poor prognosis in iCCA patients section should be corrected. The updated sentence is given below and the error found in original sentence have been highlighted in **bold typeface**.

There are several statistical data errors in the article due to author's carelessness. Although these errors do not affect the final conclusions, they should be corrected for the sake of the rigor of scientific research.

Original sentence

In multivariate regression analysis, multiple tumor number, poorer tumor diferentiation, larger tumor size, and high cZNF215 expression were independent risk factors for OS, while **the presence of MVI**, poorer tumor diferentiation, advanced TNM stage and higher cZNF215 expression were regarded as independent risk factors for RFS (Fig.1H, Supporting Table S2).

Incorrect Figure 1

**Fig. 1:**
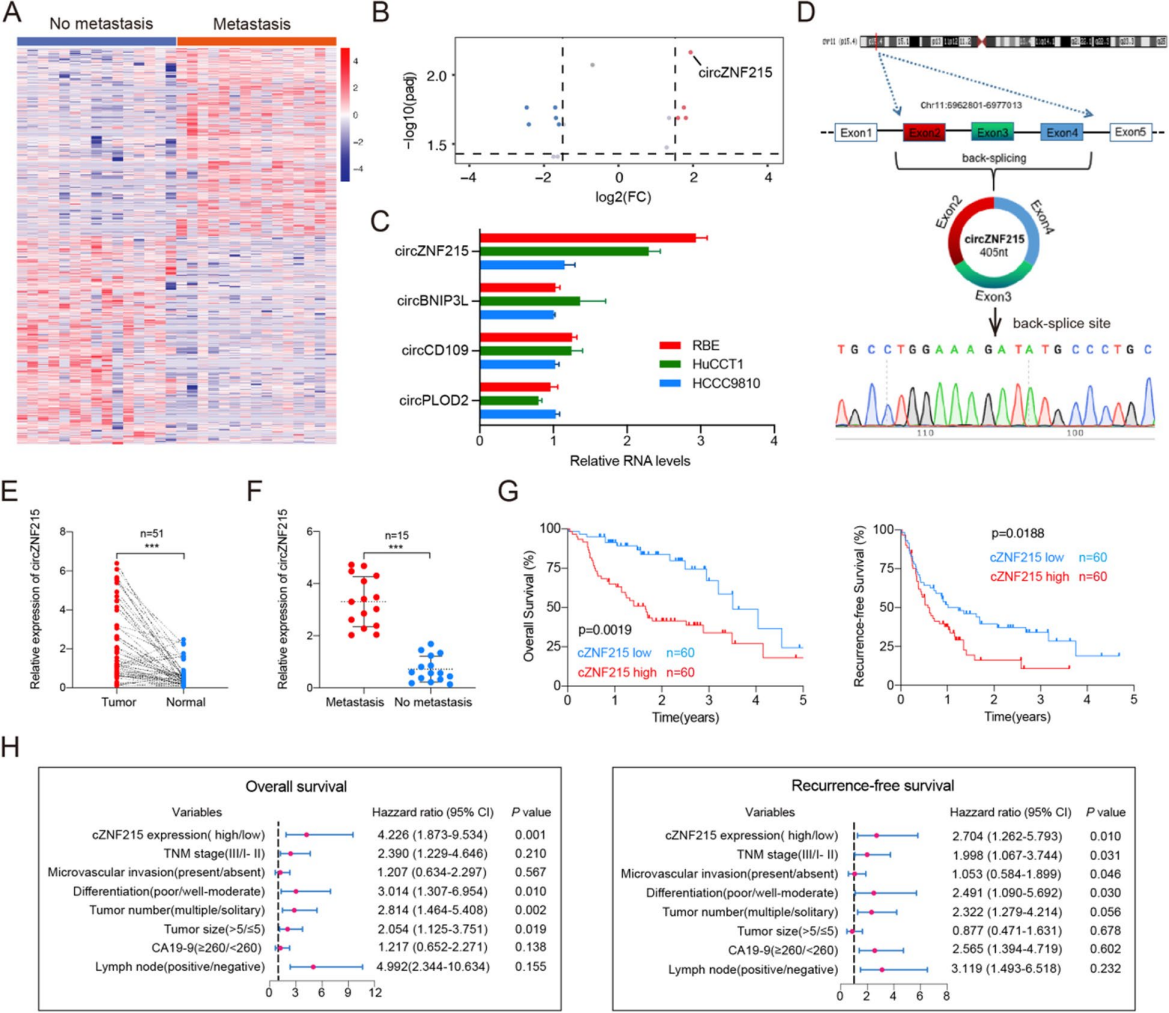
High expression of cZNF215 is correlated with iCCA metastasis and poor patient prognosis. **A** Clustered heat map of all diferentially expressed circRNAs in 15 iCCA tissues with and without extrahepatic metastasis, respectively. **B** Volcano plot compared the expression fold changes of circRNAs for iCCA tissues with extrahepatic metastases versus without extrahepatic metastases. **C** Quantitative real-time PCR analysis showed that the levels of cZNF215 expression were the highest among the 4 upregulated circRNAs in RBE, HuCCT1, and HCCC9810 cell lines. **D** Schematic illustration of cZNF215 locus with specifc primers and Sanger sequencing result of cZNF215. **E** qRT-PCR to detect the expression of cZNF215 in 51 iCCA tissues and paired normal tissues. **F** Relative RNA levels of cZNF215 in iCCA tissues (*n* = 15) with and without extrahepatic metastasis, respectively. **G** Kaplan–Meier analysis showing the association of cZNF215 expression with OS or RFS in iCCA tissues. **H** Multivariate analysis of OS and RFS prognostic indicators. Data represent means ± SD of at least three independent experiments

Updated sentence

In multivariate regression analysis, multiple tumor number, poorer tumor differentiation, larger tumor size, and high cZNF215 expression were independent risk factors for OS, while the poorer tumor differentiation, advanced TNM stage and higher cZNF215 expression were regarded as independent risk factors for RFS (Corrected Fig. 1H, Corrected Table S2).

Furthermore, Fig. 1H and Supplementary Table 2 should also be corrected. The corrected figure is given below. The original article has been corrected.

Corrected Figure 1

**Fig. 1:**
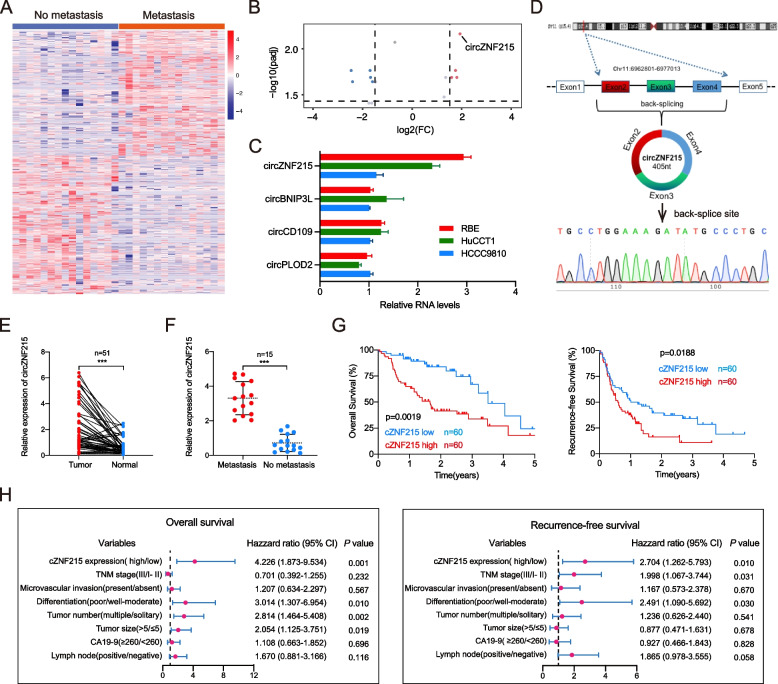
High expression of cZNF215 is correlated with iCCA metastasis and poor patient prognosis. **A** Clustered heat map of all diferentially expressed circRNAs in 15 iCCA tissues with and without extrahepatic metastasis, respectively. **B** Volcano plot compared the expression fold changes of circRNAs for iCCA tissues with extrahepatic metastases versus without extrahepatic metastases. **C** Quantitative real-time PCR analysis showed that the levels of cZNF215 expression were the highest among the 4 upregulated circRNAs in RBE, HuCCT1, and HCCC9810 cell lines. **D** Schematic illustration of cZNF215 locus with specifc primers and Sanger sequencing result of cZNF215. **E** qRT-PCR to detect the expression of cZNF215 in 51 iCCA tissues and paired normal tissues. **F** Relative RNA levels of cZNF215 in iCCA tissues (*n* = 15) with and without extrahepatic metastasis, respectively. **G** Kaplan–Meier analysis showing the association of cZNF215 expression with OS or RFS in iCCA tissues. **H** Multivariate analysis of OS and RFS prognostic indicators. Data represent means ± SD of at least three independent experiments

### Supplementary Information

**Additional file 1:** **Corrected table S2. **Multivariate analysis of several variables for OS and RFS.
