# Supplementary material for: CircZNF215 promotes tumor growth and metastasis through inactivation of the PTEN/AKT pathway in intrahepatic cholangiocarcinoma
Source: J Exp Clin Cancer Res. 2023 May 18;42:125. doi: 10.1186/s13046-023-02699-w (PMC10193609; doi:10.1186/s13046-023-02699-w)
Supplement: Supplementary file 1 — Additional file 1. Supporting Materials and Methods. [file 13046_2023_2699_MOESM1_ESM.docx]

**Supporting Materials and Methods**

**RNA sequencing**

Total RNAs were extracted from 30 iCCA tissues with ribo-zero-magnetic-kit (Epicentre) and RNase R (Epicentre), respectively. Then the ribosomal RNA and the linear RNA were removed. Next, circRNAs sequencing was applied by NEBNext® UltraTM RNA Library Prep Kit for Illumina® [1]. The samples were sequenced on an Illumina Hiseq platform. This program was performed at Novogene Co., Ltd (Beijing, China). After that, mRNA sequencing was conducted by Illumina Novaseq platform at Bio-tech ltd (Hangzhou, China). Finally, pathway analysis was carried out by gene set enrichment analysis (GSEA).

**Reverse transcription reaction and real-time quantitative PCR (RT-qPCR)**

Total RNA Isolation Kit (Foregene, China) was used to extract total RNA from iCCA cells. The first-strand cDNA was generated using HiScript II Reverse Transcriptase Kit (Vazyme, China). ChamQTM @ qPCR Master Mix (Vazyme, China) was used for RT-qPCR. The relative expression of RNA was quantitated by the 2^−ΔΔ^Ct method. The endogenous controls conclude β-actin and U6, and the primer sequences were presented in Table S3.

**RNase R treatment assay**

Trizol reagent (Invitrogen, USA) was used to extract total RNA from iCCA cells incubated with RNase R (Epicentre, 10U, 37°C, 30 min), followed by incubating for 10min at 75°C to deactivate the RNase R. RT-qPCR analysis was performed to determine the relative levels of treated RNAs.

**Actinomycin D assay**

RBE and HuCCT1 cells were equally plated in 6 wells plate. Cells were treated with actinomycin D (2μg/ml) for 0h, 4h, 8h, 12h, 24h, respectively. RT-qPCR analysis was performed to examine the expression of cZNF215 and ZNF215 mRNA.

**Subcellular RNA fractionation assay**

PARISTM Kit (Thermo, USA) was used to extract cytoplasmic and nuclear RNA fractions in accordance with its instructions, and subsequently followed by qRT-PCR analysis. U3 and β-actin were used as the endogenous nuclear control and endogenous cytoplasmic control, respectively.

**Fluorescent in situ hybridization (FISH) assay**

FISH Kit (RiboBio, China) was used for FISH assay. RiboBio (China) designed the Cy3-labled probes against cZNF215 (Table S6), 18S rRNA and U6 snRNA. A1RþMP Confocal Laser Microscope (Nikon, Japan) was used to capture images.

**Small interfering RNA transfection, plasmid transfection and lentiviral infection**

RiboBio (China) designed the cZNF215 siRNAs, which were transfected by applying GenmuteTM Reagent (SignaGen, USA) in accordance with its instructions. RiboBio (China) constructed the PTEN expression plasmid (pCDH-PTEN) and Prdx1 expression plasmid (pCDH-Prdx1) by cloning PTEN cDNA and Prdx1 cDNA into the pCDH vectors, respectively. The plasmids were transfected using GenJetTM Plus reagent (SignaGen, USA). The cells were harvested at 48h after transfection. The lentiviral vectors (OE-cZNF215, vector, sh-NC and sh-ZNF215) were synthesized by Hanbio (Shanghai, China). pHBLV-MCS-firefly luc vector was used to generate luc-expressing cell lines. Stable infection was performed according to standard protocols. The target sequences of siRNAs were presented in Table S4.

**In vitro cell proliferation assays (CCK-8, cell cycle, colony formation and EdU labelling assays)**

About 1.5 × 10^3^ cells were suspended in 100μl of complete culture media and seeded in 96-well plates. After that, 10μL of CCK-8 solution (BeyoBio, China) was added into each plate and subsequently incubated for 2 hours at 37°C. EonTM Microplate Reader (BioTek, USA) was used to detect the absorbance at 450 nm.

Cell Cycle Kit (BeyoBio, China) was used to examine cell cycle in accordance with its instructions. CytoFLEX Research Flow Cytometer analyzed the results (Beckman, USA).

Cells were seeded into triplicate wells of a 6-well plate at a density of 1.0× 10^3^ per wells and cultured for 2 weeks in colony formation assay. 4% paraformaldehyde and 0.1% crystal violet were used to fix and stain the colonies, respectively. The clone number were scanned and analyzed by using Image J software (Bethesda, USA).

EdU Apollo567 Kit (RiboBio, China) was used for EdU assay in accordance with its instructions. The proliferation rate was calculated by EdU positive rate.

**In vitro cell migration and invasion assay**

Cells were seeded in triplicate into 6-well plates for wound-healing assay. A 200μl plastic pipette tip was applied to the scratch wound when cells reached at a density of 90%. Images were captured by microscope (Zeiss, Germany) after 24h or 48h, and analyzed by Image J (Bethesda, USA).

For migration and matrigel invasion assays, 2×10^4^ or 3×10^4^ cells suspended in 500μl of serum-free 1640 medium were seeded to the upper chamber in the insert of a 24-well plate without or with matrigel, respectively. The 1640 medium containing 10% FBS was added into the lower chamber. The cells that migrated to the lower surface of the chamber were fixed and stained after incubation for 24h or 48h. Image J software was used to analyze the counts of each group.

**Western blot assay**

RIPA Lysis Buffer (BeyoBio, China) and protease and phosphatase inhibitor cocktail (Thermo, USA) were used to extract total protein from cells or tissues. Then BCA Protein Assay Kit (BeyoBio, China) was used to examine protein concentration. Proteins were separated in sodium dodecyl sulfate-polyacrylamide gel electrophoresis (SDS-PAGE) and transferred onto PVDF membranes (Millipore, USA). Next, the membranes were blocked with 5% BSA and incubated with indicated primary antibodies overnight at 4°C. Finally, the membranes were incubated with secondary antibodies. The enhanced chemiluminescent (ECL) chromogenic substrate (BeyoBio, China) was used for visualizing immunoreactivity. The intensity of signals were analyzed by ChemiDoc MP Imager System (BioRad, USA). Antibodies and reagents were presented in Table S5.

**CircRNA pull-down assay**

EZ-Magna ChIRP RNA Interactome Kit (Millipore, USA) was used for biotin-labeled circRNA pull-down assays in accordance with its instructions. RiboBio (China) designed the biotin-labelled cZNF215 probes and control probes. In brief, 20% of associated RNA-protein complexes were used to purify RNA. RT-qPCR analysis for the enrichment of cZNF215. Next, RNase A, RNase H and DNase were used to elute the remaining 80% of RNA-protein complexes. Finally, the purified proteins were analyzed by western blot or MS.

**RNA immunoprecipitation (RIP) assay**

Magna RIP^TM^ RNA-binding Protein Immunoprecipitation Kit (Millipore, USA) was used for RIP assay in accordance with its instructions. In brief, 5μg of specific antibody or normal IgG in lysis buffer (500μl) containing protease inhibitor cocktail was immobilized on magnetic beads via incubation at 37°C for 60min. Subsequently, cell lysates prepared in the complete lysis buffer were added to tubes with antibody-coated beads and incubated at 4°C overnight. Next, RNA-protein complexes were washed 6 times. 10% of coprecipitated complexes was subjected to western blot analysis to asses immunoprecipitation efficacy of antibodies. 90% of complexes were used to purify RNA.The purified RNA was analyzed by RT-qPCR.

**Co-immunoprecipitation (Co-IP) assay**

Pierce Crosslink Magnetic IP/Co-IP Kit (Thermo, USA) was used for Co-IP assay in accordance with its instructions. In brief, cells were lysed in RIPA buffer and incubated with protein A/G magnetic beads coated with 5μg of antibodies at 4°C overnight. Next, the antibody-crosslinked beads were rinsed using PBS and eluted in the elution buffer. The immune complexes were analyzed by western blot.

**Proximity ligation assay (PLA)**

Duolink in situ red starter Kit (DUO92101-1KT, Sigma-Aldrich, USA) was used to determine specific interactions between protein and protein in accordance with its instructions. In short, fluorescently labeled oligonucleotides could initiate the amplification of a Texas red reporter signal when the distance of two different proteins was less than 40nm. The fluorescent signal was analyzed by cam HRC microscope (Zeiss, Germany). Every distinct fluorescent dot represents the close proximity of two interacting proteins within the cells.

**Immunohistochemistry (IHC) assay**

For the IHC assay, iCCA specimens tissue slides were deparaffinized and rehydrated using xylene and ethanol followed by antigen retrieval with sodium citrate buffer. Samples were blocked with 5% BSA for 1 hour and incubated with primary antibodies overnight at 4°C. Horseradish peroxidase (HRP) conjugates were used for IHC staining, which was evaluated by the histochemistry score and the total score of quantitation ranges from 0 to 300.

**Immunoblotting analysis of PTEN oxidation**

The oxidative modifications of PTEN were validated by immunoblot analysis and non-reducing SDS-PAGE as described previously [2-3]. In brief, iCCA cells were lysed with NP-40 lysis buffer supplemented with 2 mM N-ethylmaleimide (NEM), which was used to alkylate the free thiol group to block the artificial oxidation. The samples were then subjected to non-reducing SDS-PAGE according to the standard protocols. For iCCA cells treated with NAC or H_2_O_2_, the cells were pretreated with 1 mM H_2_O_2_ for 10 min [3] or pretreated with 4 mM NAC for 1 h at room temperature [4].Then NEM redox immunoblot was conducted to detect the redox state of PTEN extracted from the treated cells.

**Animal studies**

In our study, male 6-week-old male BALB/c nude mice were purchased from Vital River Laboratories (Beijing, China). All mice were fed under standard pathogen-free conditions. 5×10^6^ cells were injected subcutaneously into the mice in subcutaneous xenograft models. Recorded tumor length and width once a week. Tumor volume was calculated as follows: volume (mm^3^) =0.52×a×b^2^ (a=length, b=width). Four weeks later, mice were euthanized and their tumors were isolated and weighed. In liver orthotopic-implantation models, 3×10^6^ cells were injected into the liver of each mouse. After 6 weeks, IVIS@ Lumina system (CLS, USA) was used to analyze the tumor formation and metastasis after mice intraperitoneal injected with D-luciferin (ABP, USA, 150 mg/kg). Small animal live imaging systems use two main techniques: bioluminescence and fluorescence. Bioluminescence technique uses luciferase to label iCCA cells, while fluorescence technique uses fluorescent reporter groups to label iCCA cells. In lung metastasis models, 2×10^6^ cells were injected through tail veins. After 8 weeks, IVIS@ Lumina system (CLS, USA) was used to analyze the tumor metastasis after mice intraperitoneal injected with D-luciferin. Next, mice were sacrificed after IVIS measurement, tumor tissues were excised for further detection.

To identify the effects of ipatasertib on tumor growth and metastasis, subcutaneous xenograft models, liver orthotopic-implantation models, and lung metastasis models were established using stably transfected RBE cells. Mice were randomized to receive vehicle or ipatasertib treatment and were dosed orally with or without ipatasertib once a day at 75 mg/kg for 14 days and tumor volume were monitored once 1 weeks. After establishing liver orthotopic-implantation models and lung metastasis models, mice were dosed with ipatasertib from the 3^th^ week and 5^th^ week, respectively. 2 weeks later, tumor formation and metastasis were detected by IVIS. All animal experiments in this study were approved by the Animal Ethics Committee of West China Hospital, Sichuan University.

**References**

1. Zhang Y, Zhang XO, Chen T, Xiang JF, Yin QF, Xing YH, et al. Circular intronic long noncoding RNAs. Mol Cell 2013;51:792-806

2. Zhang Y, Park J, Han SJ, Park I, Huu TN, Kim JS, Woo HA, Lee SR. The critical role of redox regulation of PTEN and peroxiredoxin III in alcoholic fatty liver. Free Radic Biol Med. 2021 Jan;162:141-148.

3. Han SJ, Zhang Y, Kim I, Chay KO, Yoon HJ, Jang DI, Yang SY, Park J, Woo HA, Park I, Lee SR. Redox regulation of the tumor suppressor PTEN by the thioredoxin system and cumene hydroperoxide. Free Radic Biol Med. 2017 Nov;112:277-286.

4. Liu X, Wang L, Cai J, Liu K, Liu M, Wang H, Zhang H. N-acetylcysteine alleviates H2O2-induced damage via regulating the redox status of intracellular antioxidants in H9c2 cells. Int J Mol Med. 2019 Jan;43(1):199-208.
