# Supplementary material for: CircZNF215 promotes tumor growth and metastasis through inactivation of the PTEN/AKT pathway in intrahepatic cholangiocarcinoma
Source: J Exp Clin Cancer Res. 2023 May 18;42:125. doi: 10.1186/s13046-023-02699-w (PMC10193609; doi:10.1186/s13046-023-02699-w)
Supplement: Supplementary file 2 — Additional file 2: Figure S1. The characteristics and localization of cZNF215. (A) qRT-PCR analysis for the expression of cZNF215 and ZNF215 mRNA in HuCCT1 and RBE cells treated with RNase R. β-actin mRNA was used as a negative control. (B) qRT-PCR analysis for the cZNF215 and ZNF215 mRNA using the template cDNA reverse-transcribed by random hexamer and oligo (dT)18 primers. (C) qRT-PCR analysis for the expression of cZNF215 and ZNF215 mRNA in HuCCT1 and RBE cells treated with Actinomycin D at indicated time points. (D) Subcellular RNA fractionation assays were conducted to determine the subcellular localization of cZNF215 and ZNF215 mRNA in HuCCT1 and RBE cells. U3 and β-actin were used as nuclear and cytoplasmic internal reference, respectively. (E) The localization of cZNF215 was further examined by FISH assays. Scale bar, 50 μm. Figure S2. cZNF215 promotes cell proliferation of iCCA cells in vitro. (A) and (B) Relative RNA levels of cZNF215 and ZNF215 mRNA after knockdown of cZNF215 in RBE and HuCCT1 cells. (C) and (D) Relative RNA levels of cZNF215 and ZNF215 mRNA after overexpression of cZNF215 in HuCCT1 and HCCC9810 cells. (E) and (F) Cell cycle analyses were performed to evaluate the effects of cZNF215 on cell cycle of iCCA cells. (G) and (H) Colony formation assays were conducted to assess the effects of cZNF215 on cell proliferation of iCCA cells. Figure S3. cZNF215 facilitates tumor growth of iCCA in vivo. (A) and (B) Effects of cZNF215 overexpression or knockdown on tumor weight in subcutaneous xenograft models. Figure S4. cZNF215 promotes iCCA proliferation and metastasis through inactivation of PTEN/AKT pathway. (A) Heatmap of differentially expressed genes of HuCCT1 cells transfected with the control and cZNF215-overexpressing lentiviral vectors. (B) and (C) The results of KEGG and GO analyses showed the main signaling pathways and the biological process affected by cZNF215 overexpression. (D) and (E) Transwell migration and matrigel invasion assays w [file 13046_2023_2699_MOESM2_ESM.docx]

**Supporting figures and figure legends**

**
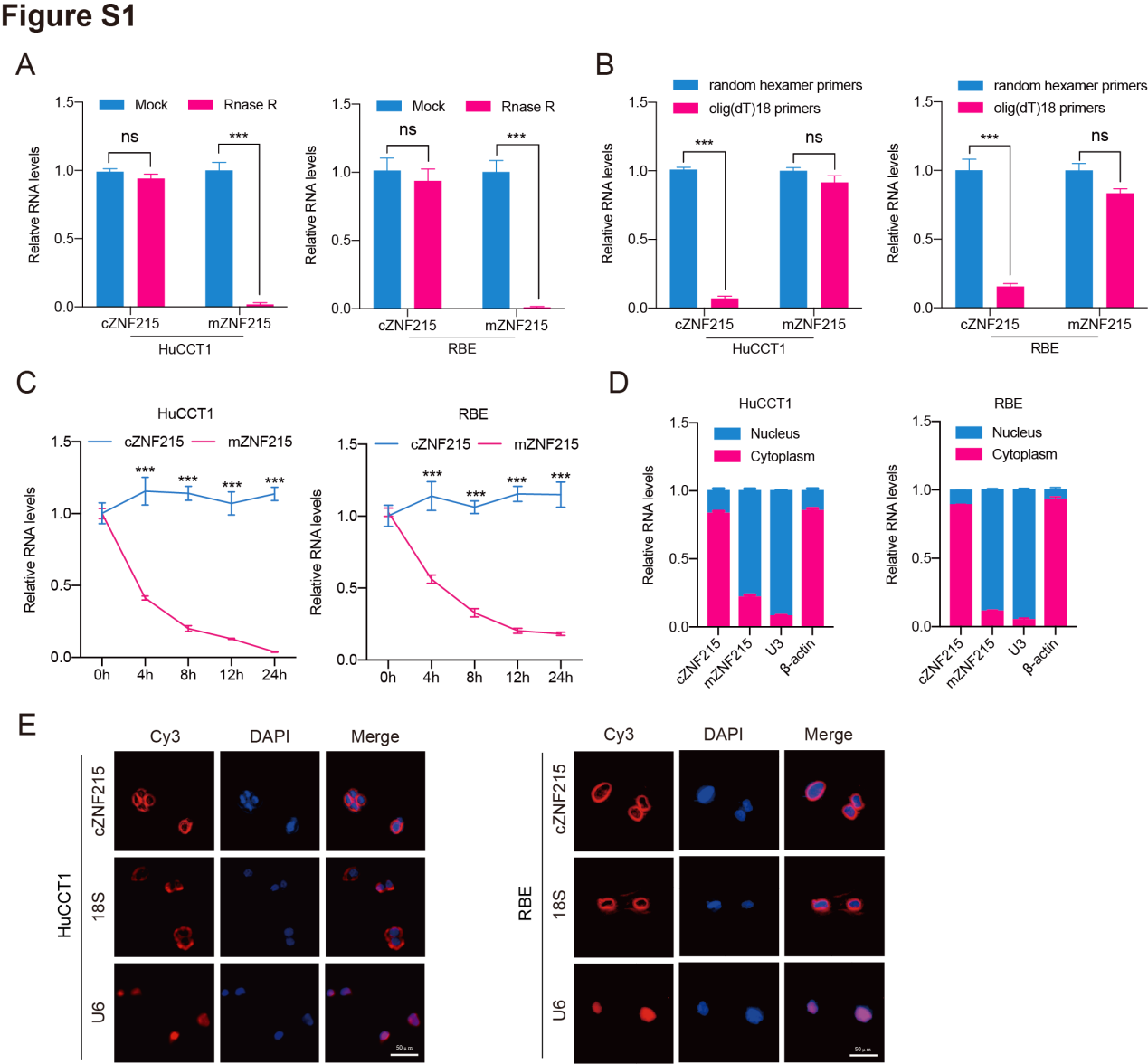
**

**Figure S1.** The characteristics and localization of cZNF215. **(A)** qRT-PCR analysis for the expression of cZNF215 and ZNF215 mRNA in HuCCT1 and RBE cells treated with RNase R. β-actin mRNA was used as a negative control. **(B)** qRT-PCR analysis for the cZNF215 and ZNF215 mRNA using the template cDNA reverse-transcribed by random hexamer and oligo (dT)18 primers. **(C)** qRT-PCR analysis for the expression of cZNF215 and ZNF215 mRNA in HuCCT1 and RBE cells treated with Actinomycin D at indicated time points. **(D)** Subcellular RNA fractionation assays were conducted to determine the subcellular localization of cZNF215 and ZNF215 mRNA in HuCCT1 and RBE cells. U3 and β-actin were used as nuclear and cytoplasmic internal reference, respectively. **(E)** The localization of cZNF215 was further examined by FISH assays. Scale bar, 50 μm.

**
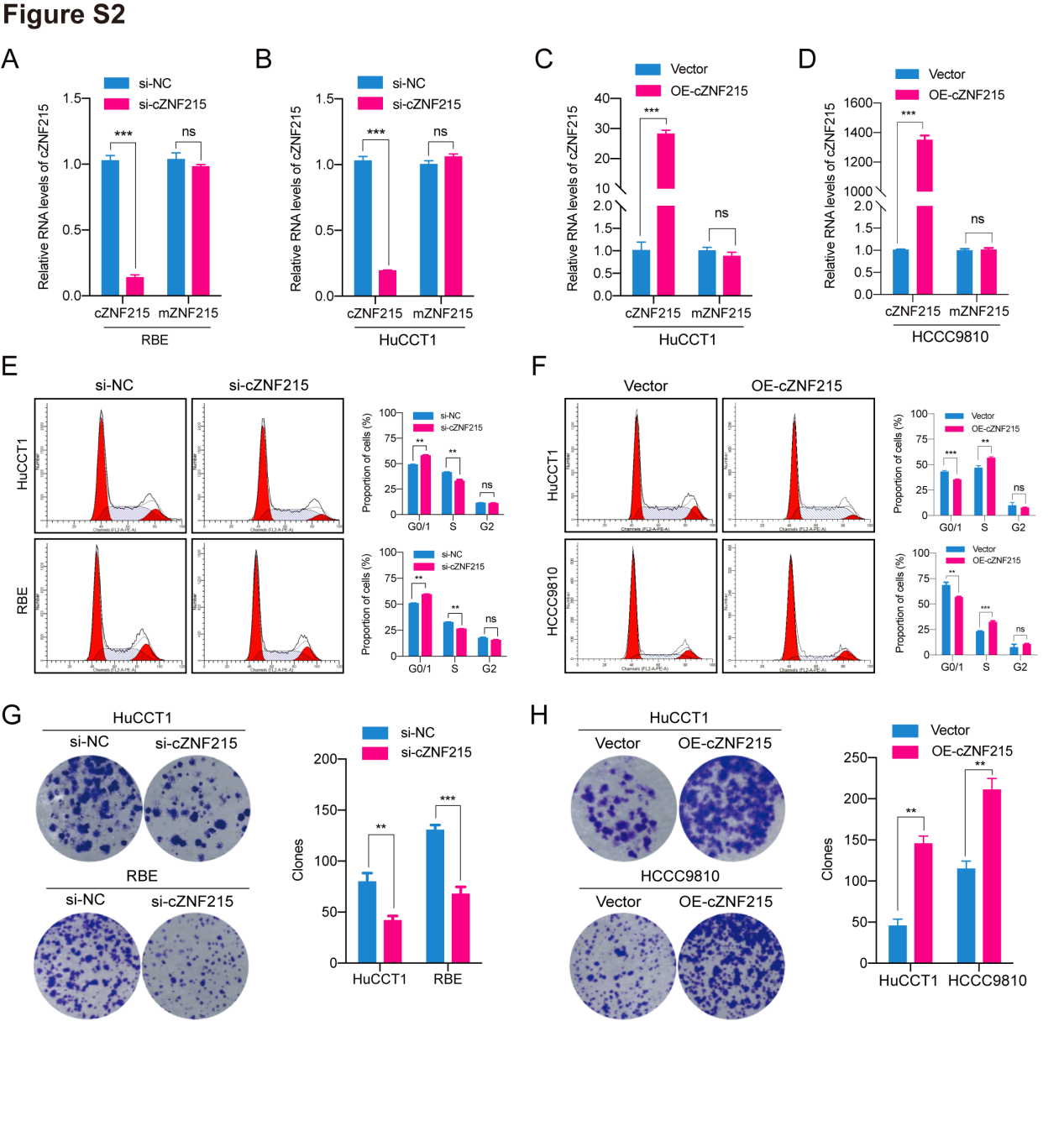
**

**Figure S2.** cZNF215 promotes cell proliferation of iCCA cells *in vitro*. **(A)** and **(B)** Relative RNA levels of cZNF215 and ZNF215 mRNA after knockdown of cZNF215 in RBE and HuCCT1 cells. **(C)** and **(D)** Relative RNA levels of cZNF215 and ZNF215 mRNA after overexpression of cZNF215 in HuCCT1 and HCCC9810 cells. **(E)** and **(F)** Cell cycle analyses were performed to evaluate the effects of cZNF215 on cell cycle of iCCA cells. **(G)** and **(H)** Colony formation assays were conducted to assess the effects of cZNF215 on cell proliferation of iCCA cells.


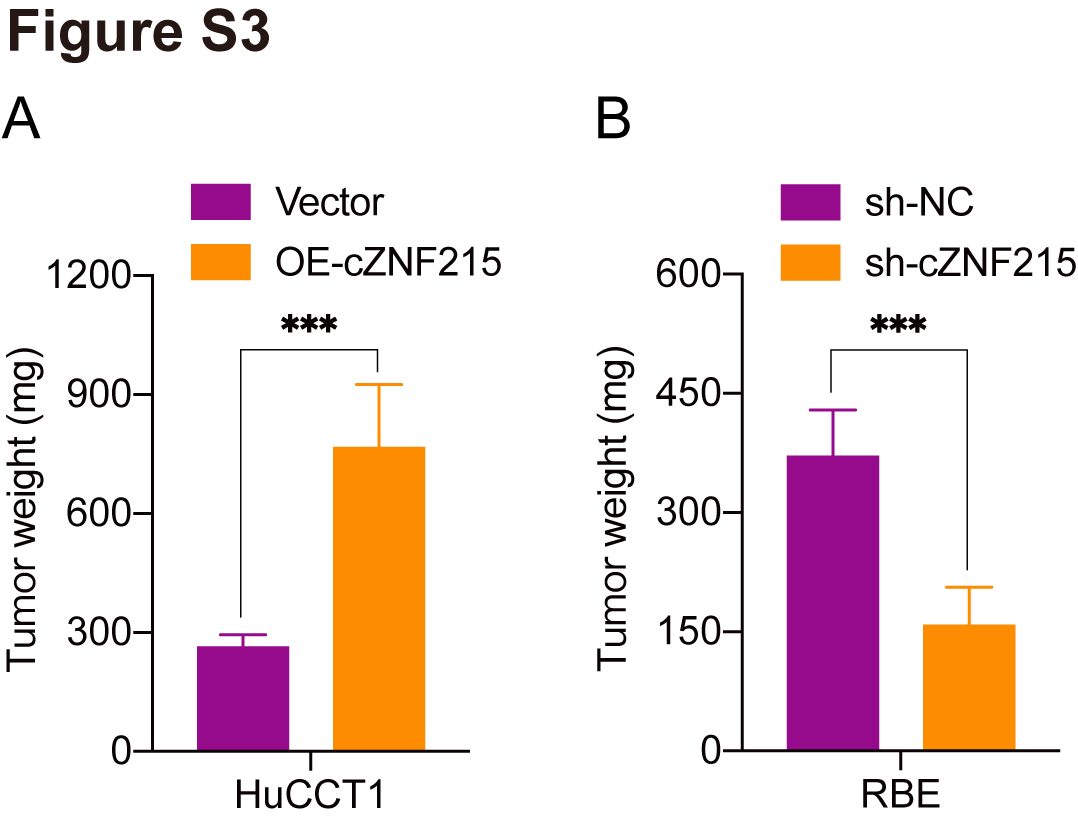


**Figure S3.** cZNF215 facilitates tumor growth of iCCA *in vivo*. **(A)** and **(B)** Effects of cZNF215 overexpression or knockdown on tumor weight in subcutaneous xenograft models.


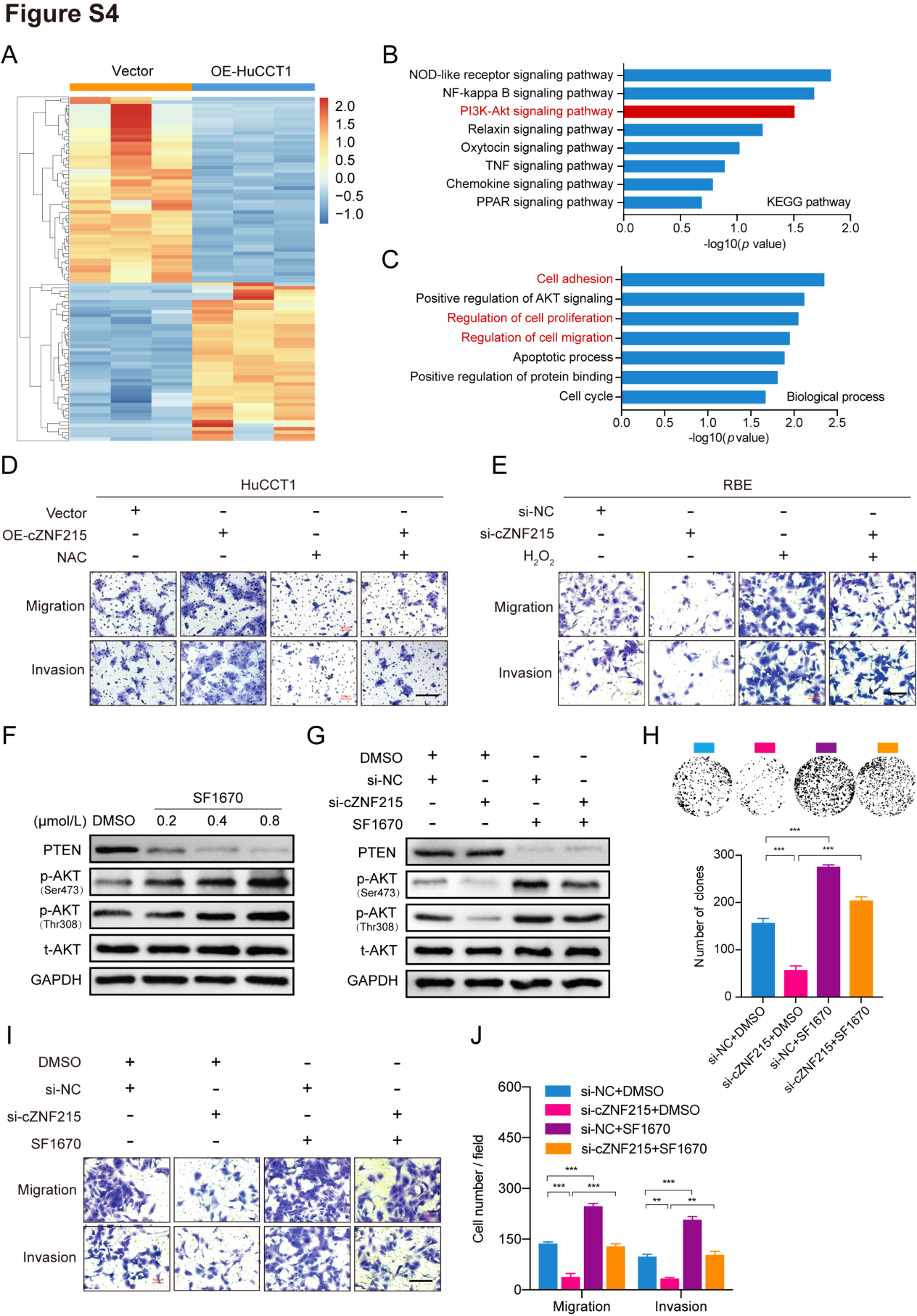


**Figure S4.** cZNF215 promotes iCCA proliferation and metastasis through inactivation of PTEN/AKT pathway. **(A)** Heatmap of differentially expressed genes of HuCCT1 cells transfected with the control and cZNF215-overexpressing lentiviral vectors. **(B)** and **(C)** The results of KEGG and GO analyses showed the main signaling pathways and the biological process affected by cZNF215 overexpression. **(D)** and **(E)** Transwell migration and matrigel invasion assays were performed in HuCCT1 cells treated with indicated vectors and NAC (an antioxidant), or RBE cells treated with cZNF215 siRNAs and H_2_O_2_. **(F)** Western blot analysis for p-AKT^Ser473/Thr308^ and t-AKT expression using protein extracts from RBE cells treated with SF1670 (a PTEN inhibitor) for 24h. **(G)** Western blot assay for PTEN, p-AKT^Ser473/Thr308^ and t-AKT protein levels in RBE cells transfected with cZNF215 siRNA or treated with SF1670 (0.4μM). GAPDH served as the internal reference. **(H)** Colony formation assays were conducted to examine colony formation capacity of RBE cells. **(I)** and **(J)** Transwell assays were performed to determine the migration and invasion capacity of RBE cells. Scale bars, 200 μm.


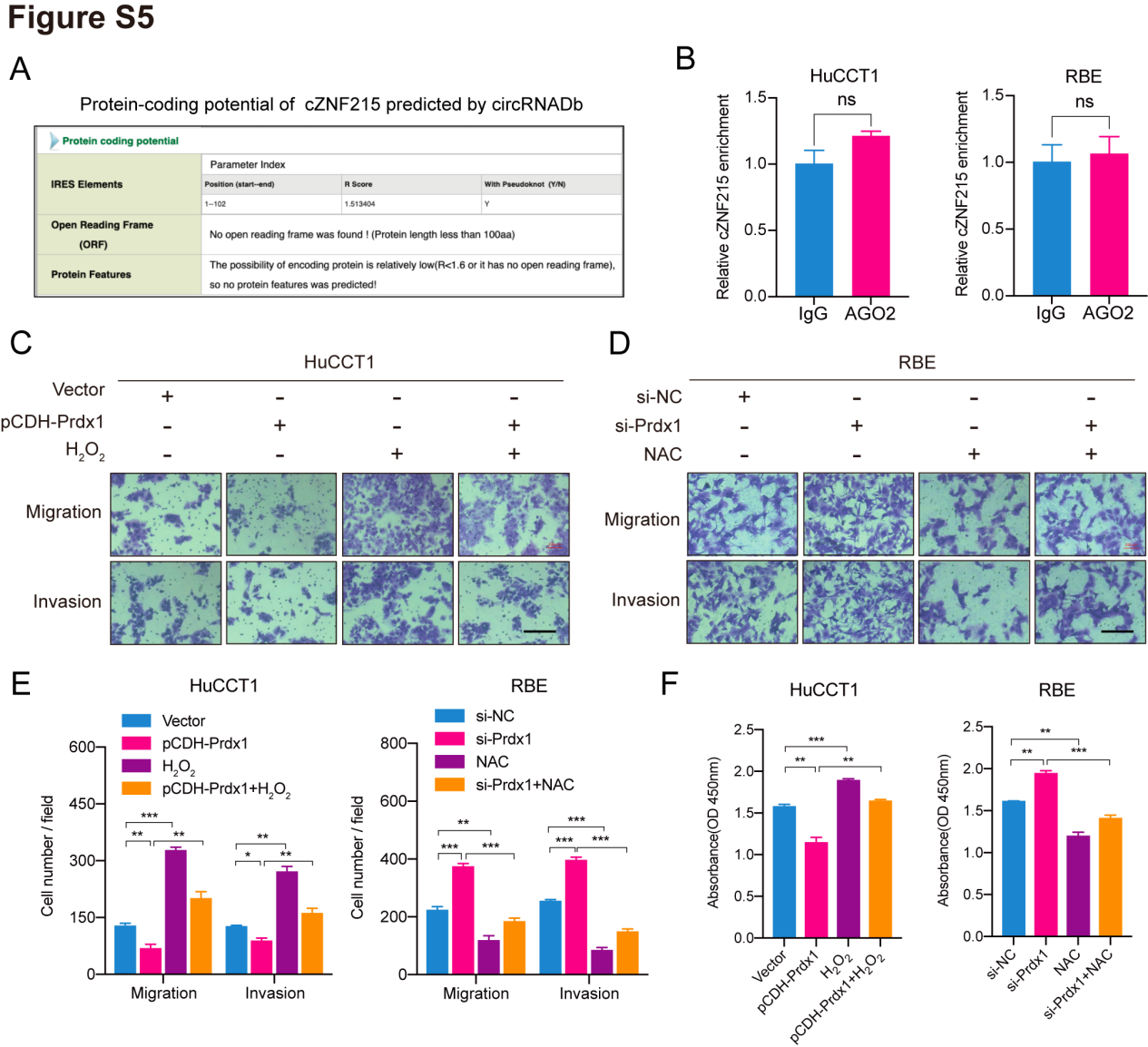


**Figure S5.** PRDX1 is involved in cZNF215-mediated PTEN/AKT pathway inactivation by interacting with cZNF215. **(A)** Prediction of protein-coding potential of cZNF215 using the circRNADb algorithm. **(B)** RIP assays were conducted in HuCCT1 and RBE cells to determine the interaction between cZNF215 and AGO2. **(C, D and E)** Transwell migration and matrigel invasion assays were conducted in HuCCT1 cells treated with Prdx1 (an antioxidant enzyme) vectors and H_2_O_2_, or RBE cells treated with Prdx1 siRNAs and NAC. **(F)** CCK8 assays showed the proliferation ability of HuCCT1 and RBE cells. Scale bars, 200 μm.


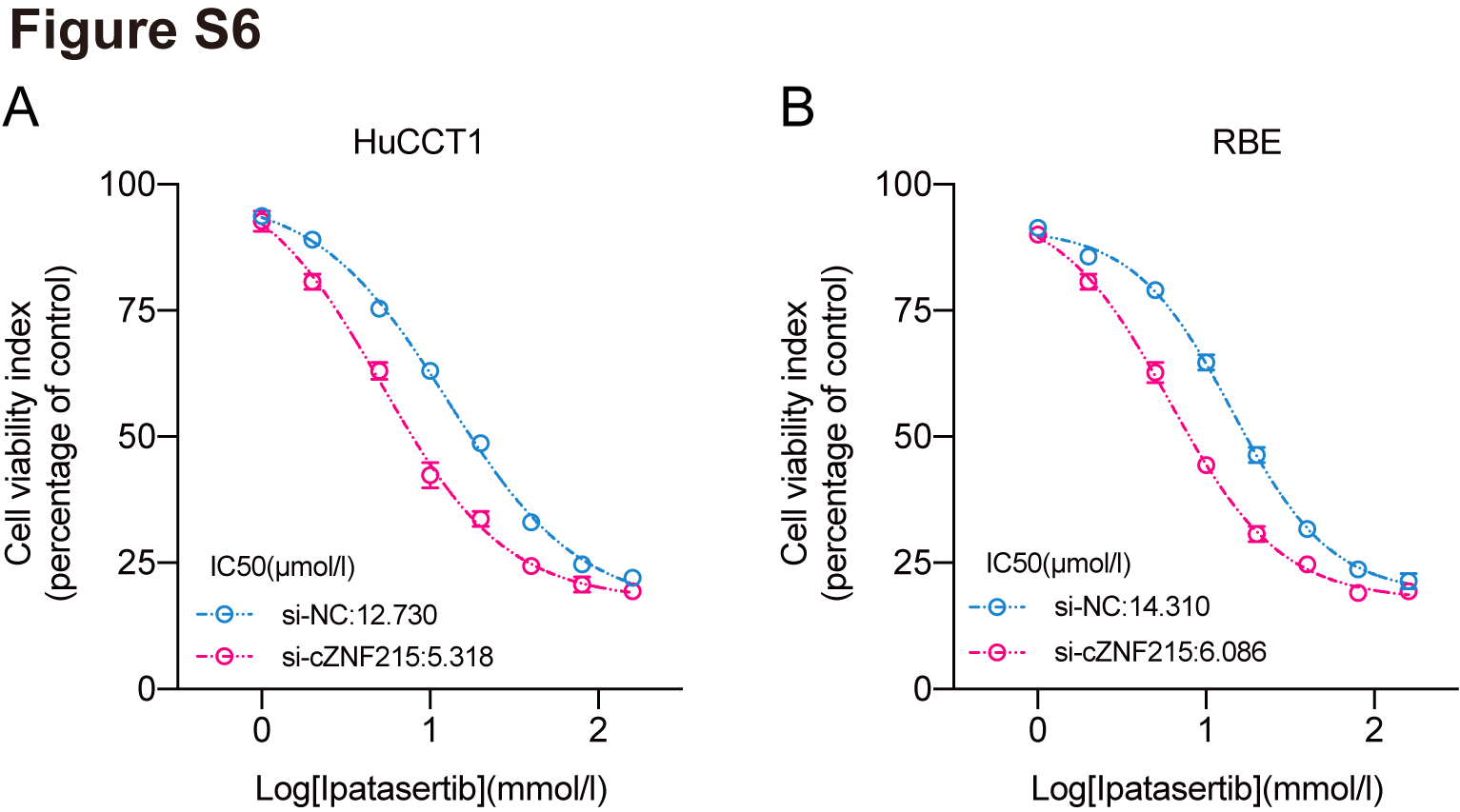


**Figure S6.** cZNF215 serves as a potential therapeutic target of iCCA. **(A)** and **(B)** Ipatasertib was used to incubate with HuCCT1 or RBE cells for 72h and the IC50 value was calculated by CCK-8 assays.


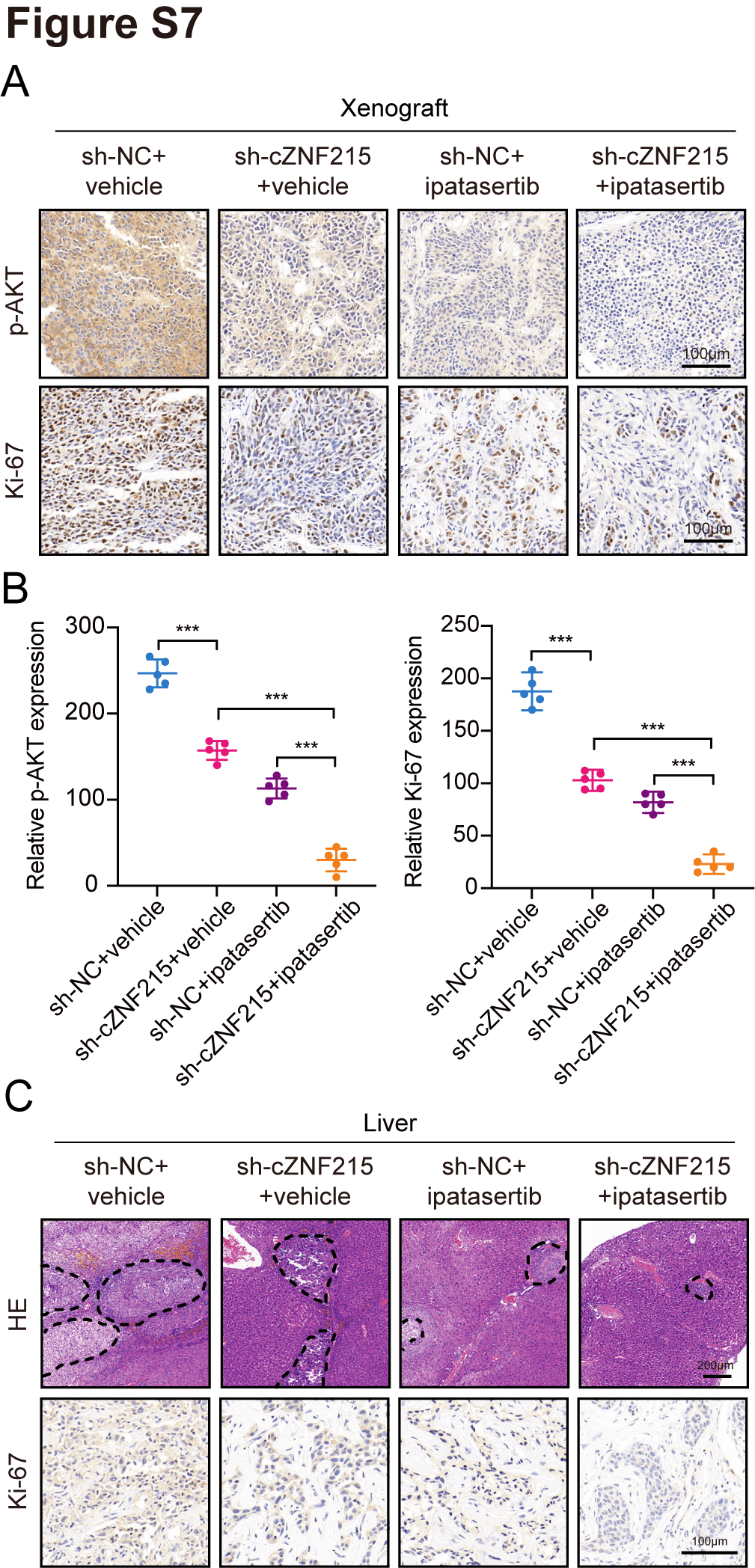


**Figure S7.** Silencing of cZNF215 enhances antitumor effect of ipatasertib *in vivo*. **(A)** and **(B)** Representative IHC staining of p-AKT^Ser473/Thr308^ and Ki-67, and quantitative analysis of p-AKT^Ser473/Thr308^ and Ki-67 staining results in xenografts. Scale bars, 100 μm. **(C)** Top, representative HE staining of metastatic foci in the livers. Scale bars, 200 μm. Bottom, representative IHC staining of Ki-67 in the livers. Scale bars, 100 μm.
