# Supplementary material for: CircZNF215 promotes tumor growth and metastasis through inactivation of the PTEN/AKT pathway in intrahepatic cholangiocarcinoma
Source: J Exp Clin Cancer Res. 2023 May 18;42:125. doi: 10.1186/s13046-023-02699-w (PMC10193609; doi:10.1186/s13046-023-02699-w)
Supplement: Supplementary file 3 — Additional file 3: Table S1. Univariate analysisof several variables for OS and RFS. Table S2. Multivariate analysis ofseveral variables for OS and RFS. Table S3. The primers sequences wereas follows. Table S4. The target sequences of siRNAs were as follows. Table S5. Antibodies and reagents were as follows. Table S6. FISH probes used in this study. [file 13046_2023_2699_MOESM3_ESM.docx]

**Supporting tables**

**Table S1. Univariate analysis of several variables for OS and RFS**

| Variables | Overall survival | | Recurrence-free survival | |
| --- | --- | --- | --- | --- |
|  | Hazzard ratio (95% CI) | *P* value | Hazzard ratio (95% CI) | *P* value |
| Age, year, >60/≤60 | 0.763 (0.415-1.403) | 0.384 | 1.329 (0.725-2.435) | 0.358 |
| Gender, male/female | 0.949 (0.526-1.711) | 0.862 | 1.000 (0.564-1.775) | 0.999 |
| Ascites, present/absent | 1.060 (0.496-2.267) | 0.880 | 1.947 (0.912-4.157) | 0.085 |
| Hepatolithiasis, present/absent | 1.494 (0.206-10.855) | 0.692 | 1.106 (0.153-8.019) | 0.921 |
| CA19-9, ≥260/<260 | 1.916 (1.109-3.310) | **0.020** | 1.937 (1.123-3.464) | **0.018** |
| Tumor size (cm) >5/≤5 | 1.983 (1.158-3.394) | **0.013** | 1.838 (1.068-3.163) | **0.028** |
| Tumor number, multiple/solitary | 2.438 (1.419-4.188) | **0.001** | 2.138 (1.255-3.640) | **0.005** |
| Differentiation, poor/well-moderate | 3.380 (1.515-7.545) | **0.003** | 3.279 (1.476-7.283) | **0.004** |
| MVI, present/absent | 2.353 (1.308-4.232) | **0.004** | 1.874 (1.068-3.289) | **0.029** |
| Lymph node, positive/negative | 6.879 (3.757-12.593) | **<0.001** | 5.189 (2.904-9.271) | **<0.001** |
| Cirrhosis, with/without | 1.990 (0.891-4.445) | 0.093 | 1.947 (0.912-4.157) | 0.085 |
| TNM stage, III/I- II | 3.844 (2.054-7.195) | **<0.001** | 3.053 (1.718-5.425) | **<0.001** |
| cZNF215 expression, high/low | 4.837 (2.702-8.659) | **<0.001** | 3.798 (2.146-6.722) | **<0.001** |
| Chronic viral hepatitis B, present/absent | 1.312 (0.771-2.233) | 0.317 | 1.277 (0.641-2.545) | 0.487 |

CI, confidence interval; MVI, microvascular invasion; TNM, tumor-node-metastasis.

**Corrected table S2. Multivariate analysis of several variables for OS and RFS**

| Variables | Overall survival | | Recurrence-free survival | |
| --- | --- | --- | --- | --- |
|  | Hazzard ratio (95% CI) | *P* value | Hazzard ratio (95% CI) | *P* value |
| CA19-9, ≥260/<260 | - | ns | - | ns |
| Tumor size (cm) >5/≤5 | 2.054 (1.125-3.751) | 0.019 | - | ns |
| Tumor number, multiple/solitary | 2.814 (1.464-5.408) | 0.002 | - | ns |
| Differentiation, poor/well-moderate | 3.014 (1.307-6.954) | 0.010 | 2.491 (1.090-5.692) | 0.030 |
| MVI, present/absent | - | ns | - | ns |
| Lymph node, positive/negative | - | ns | - | ns |
| TNM stage, III/I- II | - | ns | 1.998 (1.067-3.744) | 0.031 |
| cZNF215 expression, high/low | 4.226 (1.873-9.534) | 0.001 | 2.704 (1.262-5.793) | 0.010 |

iCCA, intrahepatic cholangiocarcinoma; MVI, microvascular invasion; CI, confidence interval; TNM, tumor-node-metastasis; ns, no significance.

In multivariate regression analysis, multiple tumor number, poorer tumor differentiation, larger tumor size, and high cZNF215 expression were independent risk factors for OS, while the poorer tumor differentiation, advanced TNM stage and higher cZNF215 expression were regarded as independent risk factors for RFS (Corrected Fig.1H, Corrected Table S2).

**Table S3. The primers sequences were as follows.**

| **Name** | **Forward sequence** | **Reverse sequence** | **Supplier** |
| --- | --- | --- | --- |
| cZNF215 | TCAGATGCCTGGAAAGATATGC | CCCCACTCTTCCTTGCTGAA | TsingKe |
| mZNF215 | TGTGGAGTTCGCACACTCTC | GGCAATGGTGGGAACAAACC | TsingKe |
| U6 | TCGGCAGCACATATACTAAAATTGG | ACGAATTTGCGTGTCATCCT | TsingKe |
| cCD109 | AACAGTGGTTGTCACAACAAAGT | AGTGTCTTAAAAGAGCATTCACTTGA | TsingKe |
| cPLOD2 | TGGAAATGGACCCACCAAGA | CCTTGACCAAGGACCTTCACA | TsingKe |
| cBNIP3L | CTTTGGGGCTAGGGTTCCTG | TCTCCATGTCTCCATTGTGGA | TsingKe |
| U3 | TTCTCTGAGCGTGTAGAGCACCGA | GATCATCAATGGCTGACGGCAGTT | TsingKe |
| β-actin | CTCGCCTTTGCCGATCC | TCTCCATGTCGTCCCAGTTG | TsingKe |

**Table S4. The target sequences of siRNAs were as follows.**

| **Name** | **Forward sequence** | **Reverse sequence** | **Supplier** |
| --- | --- | --- | --- |
| si-cZNF215 | GCCUGGAAAGAUAUGCCCUTT | AGGGCAUAUCUUUCCAGGCTT | Ribobio |
| si-PTEN | GAUGAGACUUUGAGACUAGUU | AACUAGUCUCAAAGUCUCAUC | Ribobio |
| si-PRDX1 | CCAUGAACAUUCCUUUGGUAU | AUACCAAAGGAAUGUUCAUGG | Ribobio |

**Table S5. Antibodies and reagents were as follows.**

| **Name** | **Supplier** | **Cat no.** |
| --- | --- | --- |
| PTEN Polyclonal Antibody | Proteintech | 22034-1-AP |
| Phospho-Akt (Ser473) (D9E) Rabbit mAb | CST | 4060 |
| Phospho-Akt (Thr308) (D25E6) Rabbit mAb | CST | 13038 |
| Akt (pan) (C67E7) Rabbit mAb | CST | 4691 |
| Mouse anti PRDX1 monoclonal antibody | Proteintech | 66820-1-Ig |
| GAPDH (8A3) Mouse mAb | ZEN-bioscience | 250133 |
| Anti-Flag Rabbit mAb | CST | 14793S |
| Goat anti-Mouse IgG (H&L)（HRP conjugate） | ZEN-bioscience | 511103 |
| Goat anti-Rabbit IgG (H&L)（HRP conjugate） | ZEN-bioscience | 511203 |
| SF1670 | MCE | HY-15842 |
| Ipatasertib | MCE | HY-15186 |
| N-Ethylmaleimide | Sigma-Aldrich | E3876 |

**Table S6. FISH probes used in this study.**

| Name | Target sequence | Supplier |
| --- | --- | --- |
| cZNF215 biotinylated probe | GCATATCTTTCCAGGCATCT-/3bio/ | Ribobio |
| Control biotinylated probe | GTTTACCGCGATTACCTCTA-/3bio/ | Ribobio |
